# Biotinylated Tn5 transposase‐mediated CUT&Tag efficiently profiles transcription factor‐DNA interactions in plants

**DOI:** 10.1111/pbi.14029

**Published:** 2023-03-02

**Authors:** Xiao‐Yuan Tao, Xue‐Ying Guan, Gao‐Jie Hong, Yu‐Qing He, Su‐Juan Li, Shou‐Li Feng, Jian Wang, Guang Chen, Fei Xu, Jia‐Wei Wang, Sheng‐Chun Xu

**Affiliations:** ^1^ Central Laboratory, State Key Laboratory for Managing Biotic and Chemical Threats to the Quality and Safety of Agro‐products Zhejiang Academy of Agricultural Sciences Hangzhou China; ^2^ College of Agriculture and Biotechnology Zhejiang University Hangzhou China; ^3^ Institute of Virology and Biotechnology Zhejiang Academy of Agricultural Sciences Hangzhou China; ^4^ National Key Laboratory of Plant Molecular Genetics (NKLPMG), CAS Center for Excellence in Molecular Plant Sciences (CEMPS), Institute of Plant Physiology and Ecology (SIPPE) Chinese Academy of Sciences (CAS) Shanghai China; ^5^ Xianghu Laboratory Hangzhou China

**Keywords:** AtSPL9, B‐CUT&Tag, defence, flowering, *OsPHR2*, Tn5

## Abstract

In contrast to CUT&Tag approaches for profiling bulk histone modifications, current CUT&Tag methods for analysing specific transcription factor (TF)‐DNA interactions remain technically challenging due to TFs having relatively low abundance. Moreover, an efficient CUT&Tag strategy for plant TFs is not yet available. Here, we first applied biotinylated Tn5 transposase‐mediated CUT&Tag (B‐CUT&Tag) to produce high‐quality libraries for interrogating TF‐DNA interactions. B‐CUT&Tag combines streptavidin‐biotin‐based DNA purification with routine CUT&Tag, optimizing the removal of large amounts of intact chromatin not targeted by specific TFs. The biotinylated chromatin fragments are then purified for construction of deep sequencing libraries or qPCR analysis. We applied B‐CUT&Tag to probe genome‐wide DNA targets of Squamosa promoter‐binding‐like protein 9 (SPL9), a well‐established TF in *Arabidopsis*; the resulting profiles were efficient and consistent in demonstrating its well‐established target genes in juvenile‐adult transition/flowering, trichome development, flavonoid biosynthesis, wax synthesis and branching. Interestingly, our results indicate functions of AtSPL9 in modulating growth‐defence trade‐offs. In addition, we established a method for applying qPCR after CUT&Tag (B‐CUT&Tag‐qPCR) and successfully validated the binding of SPL9 in *Arabidopsis* and PHR2 in rice. Our study thus provides a convenient and highly efficient CUT&Tag strategy for profiling TF‐chromatin interactions that is widely applicable to the annotation of *cis*‐regulatory elements for crop improvement.

## Introduction

The study of protein‐chromatin interactions characterizes the dynamic binding of transcription factors or chromatin architecture factors to cis‐regulatory elements of the genome, which account for the spatiotemporal control of gene expression (Spitz and Furlong, 2012). For decades, the traditional assay for determining the occupancy of individual TFs has been chromatin immunoprecipitation (ChIP) coupled with either deep sequencing (ChIP‐seq) or target‐specific quantitative PCR (ChIP‐qPCR). In these immunoprecipitation‐based methods, a protein of interest is enriched along with its associated nascent DNA fragments (Furey, 2012). However, ChIP experiments suffer from need for high sample input, low signal‐to‐noise ratio and inconsistency of signals (Bartosovic *et al*., 2021). Cleavage Under Targets and Tagmentation (CUT&Tag) is an alternative strategy for epigenomic profiling, in which the target chromatin protein is bound in situ by the specific antibody, which then tethers the protein A/G‐Tn5 transposase fusion protein. The pA/G‐Tn5 transposase is activated in the presence of Mg^2+^ and acts to ‘cut‐and‐paste’ the target chromatin, resulting as the fragmented target DNA tagging with adaptor sequences, that is tagmentation reaction (Kaya‐Okur *et al*., 2019). In CUT&Tag for animal cells, the cell membrane is permeabilized with digitonin (a non‐ionic detergent) to allow the antibodies and pA‐Tn5 to diffuse into the cell. While for plant CUT&Tag, as the presence of cell wall in plant cells cannot be permeabilized effectively by digitonin, the plant intact nuclei need to be isolated for reaction. CUT&Tag‐seq outperforms ChIP in its simplicity, low dependence on devices/kits, low input requirement and high signal‐to‐noise ratio (Kaya‐Okur *et al*., 2020).

While CUT&Tag approaches for profiling histone modifications have seen good success (Bartosovic *et al*., 2021; Kaya‐Okur *et al*., 2020; Tao *et al*., 2020; Wu *et al*., 2021), analysing specific transcription factor (TF)‐DNA interactions with CUT&Tag remains technically challenging due to the relatively low abundances of TFs. To date, only a few general chromatin architecture factors present in high abundance (CTCF and Rad21) and limited number of TFs (Olig2, Pax6) have been successfully profiled using CUT&Tag in mammalian cells (Bartosovic *et al*., 2021; Kaya‐Okur *et al*., 2019; Qiao *et al*., 2022). An efficient CUT&Tag strategy with successful application to plant TFs is not currently available. In addition, in this enzyme‐tethering method, target chromatin sites are fragmented by the *in situ* ‘cut‐and‐paste’ action of Tn5 transposase (Reznikoff, 2003), which means that intact chromatin not recognized by the antibody remains present after reaction. Thus, it is not currently possible to apply qPCR after standard CUT&Tag.

Here in this work, we provide for the first time a biotinylated Tn5 transposase‐mediated CUT&Tag (B‐CUT&Tag) strategies to produce a high‐quality library for TF‐DNA interactions. We focused on Squamosa promoter‐binding‐like protein 9 (SPL9) in *Arabidopsis*, which functions in juvenile‐adult transition/flowering (Wang *et al*., 2009), trichome development (Yu *et al*., 2010), flavonoid biosynthesis (Gou *et al*., 2011), wax synthesis (Li *et al*., 2019), branching (Xie *et al*., 2020) and gibberellin (GA) and jasmonate (JA) responses (Mao *et al*., 2017; Zhang *et al*., 2020). We were able to profile AtSPL9 occupancy in *Arabidopsis* and to confirm the method's accuracy and consistency by its good recapitulation of the well‐established target genes of AtSPL9, which were previously confirmed by multiple *in vitro* and *in vivo* assays including reporter gene activity assays involving mutation of the core ‘GTAC’ motif in promoters (Gou *et al*., 2011; Yu *et al*., 2010), EMSA assays (Li *et al*., 2019) and ChIP‐qPCR (Gou *et al*., 2011; Yu *et al*., 2010). We further combined B‐CUT&Tag and mRNA‐seq data to obtain multiple high‐confidence candidate target genes of AtSPL9 with involvement in plant defence responses. Finally, we applied an optimized B‐CUT&Tag‐qPCR procedure to confirm binding of TFs including AtSPL9 and OsPHR2 to their specific targets. Taken together, this study provides a method to effectively study the epigenetic regulation of TFs in plants.

## Results

### Working principle of B‐CUT&Tag

**Figure 1:**
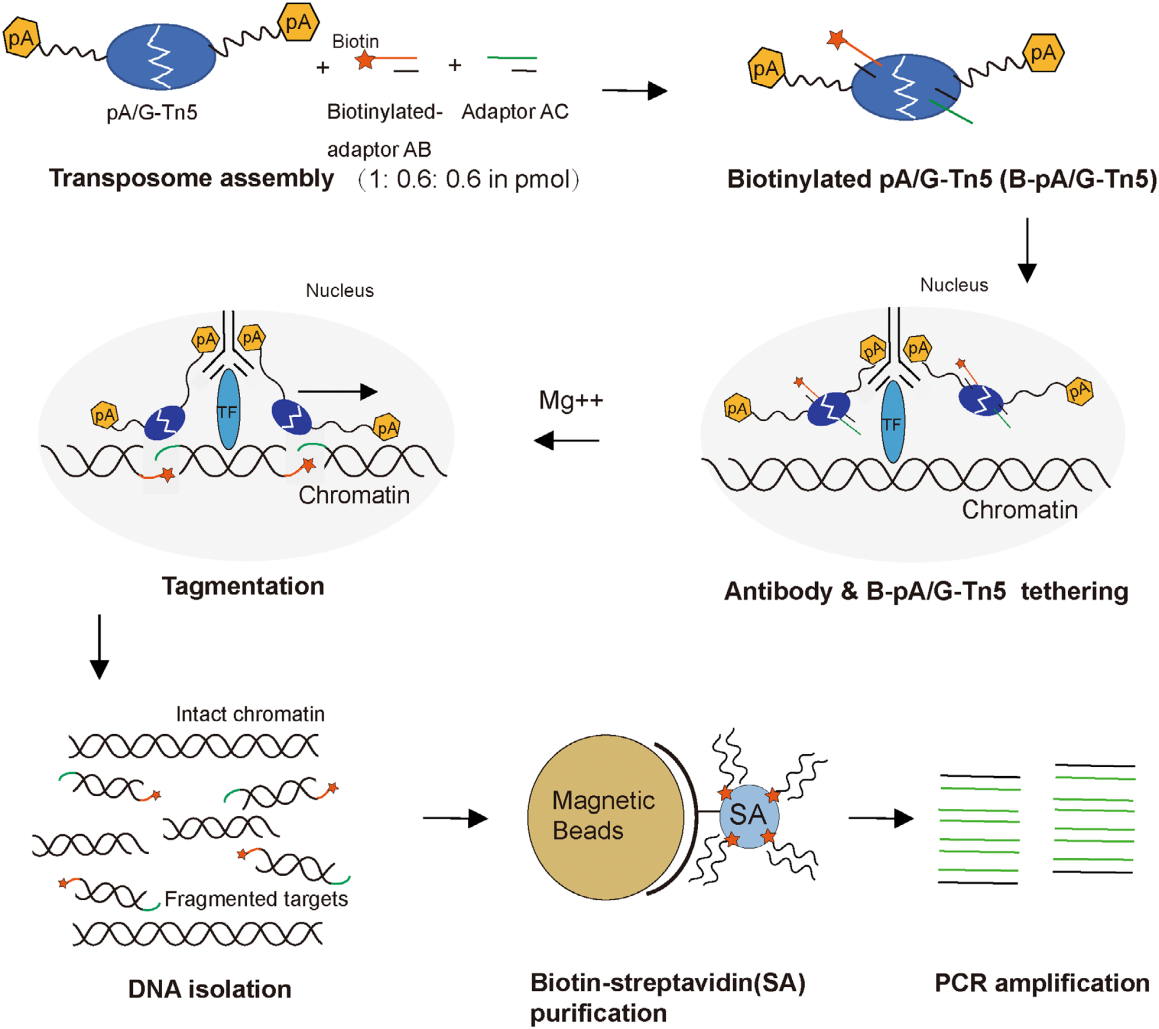
Working principle of biotinylated adaptor‐mediated CUT&Tag (B‐CUT&Tag). pA/G‐Tn5 transposase was complexed with biotinylated adaptor AB and non‐biotinylated adaptor AC at 1:0.6:0.6 ratio to form the Tn5 transposase homo‐dimer. In the nuclei, TF is bound in situ by the specific antibody, which then tethers the biotinylated pA/G‐Tn5 transposase (B‐pA/G‐Tn5). The B‐pA/G‐Tn5 is activated in the presence of Mg^2+^ and performs tagmentation reaction, resulting as the biotinylated fragments from the target sites, which was purified using the biotin‐streptavidin (biotin‐SA) magnetic beads and then subject to PCR amplification.

Our B‐CUT&Tag approach builds on biotinylated Tn5 transposase‐mediated tagmentation and biotin‐streptavidin‐based purification. In principle, two Tn5 transposase monomers were complexed with adaptors containing the 19 bp Tn5 mosaic end (ME) transposon sequence (Reznikoff, 2003) and the 14/15 bp 5′ adaptor sequence compatible with Illumina paired‐end sequencing (adaptor AB: adaptor AC at 1:1 ratio, refer to Methods for details) to form the Tn5 transposase homo‐dimer (Xu *et al*., 2021), in which Primer B in adaptor AB were 5′ biotin‐triethylene glycol spacer (TEG) modified (Table S1). The intact nuclei were isolated and applied for plant B‐CUT&Tag. After antibody binding (including a primary antibody and a secondary antibody, refer to Method part for details), pA/G‐Tn5 tethering and tagmentation reaction, the biotinylated adaptor was incorporated into the target chromatin sites, giving rise to biotinylated chromatin fragments. The total DNA was then extracted and the biotinylated DNA fragments were purified using a biotin‐streptavidin (biotin‐SA) magnetic beads purification system, resulting in the removal of large amounts of intact chromatin not targeted by the selected TF. The resultant biotinylated DNA fragments were used for library construction and deep sequencing (B‐CUT&Tag‐seq) or subjected to quantitative PCR (B‐CUT&Tag‐qPCR) (Figure 1, refer to Methods for details).

### Formaldehyde cross‐linking pre‐treatment is compatible with CUT&Tag

TFs display transient, dynamic and unstable properties when bound to their target DNA (Swift and Coruzzi, 2017). Thus, the unfixed/native conditions employed in CUT&Tag for abundant histone markers are not suitable when assaying TFs. To overcome this problem, chromatin cross‐linking is included as a mandatory step to preserve the protein‐DNA interactions. We first asked whether formaldehyde cross‐linked nuclei can be used for CUT&Tag and compared native CUT&Tag and cross‐linked CUT&Tag (X‐CUT&Tag) at profiling H3K27me3 landscape using nuclei isolated from leaves of 30‐day‐old cotton seedlings. X‐CUT&Tag was performed using isolated nuclei pre‐treated with 1% formaldehyde for 5 min. The results showed that X‐CUT&Tag generated H3K27me3 signals consistent with native CUT&Tag (Figure S1). H3K4me3 profiling using the same nuclei from cotton leaves is provided for comparison. From the results, a trend was discovered that chromatin with higher degrees of H3K27me3 modification showed lower H3K4me3 levels (e.g. genes in cluster 1) and vice versa (genes in cluster 2) (Figure S1), indicating that CUT&Tag is applicable not only for detecting activating chromatin markers but also silencing chromatin markers. A lowered FRiP value in H3K27me3 X‐CUT&Tag sample was found compared with native CUT&Tag (Figure S1), which may be resulted from the overcross‐linking of the nuclei. About 75% peaks were shared between X‐CUT&Tag and native CUT&Tag (Figure S1), indicating that the anti‐H3K27me3 antibody and Tn5 transposase can enter the cross‐linked nuclei efficiently. Thus, formaldehyde cross‐linked nuclei are compatible with CUT&Tag.

### B‐CUT&Tag generates consistent profiles compared with routine CUT&Tag under low input of nuclei

**Figure 2:**
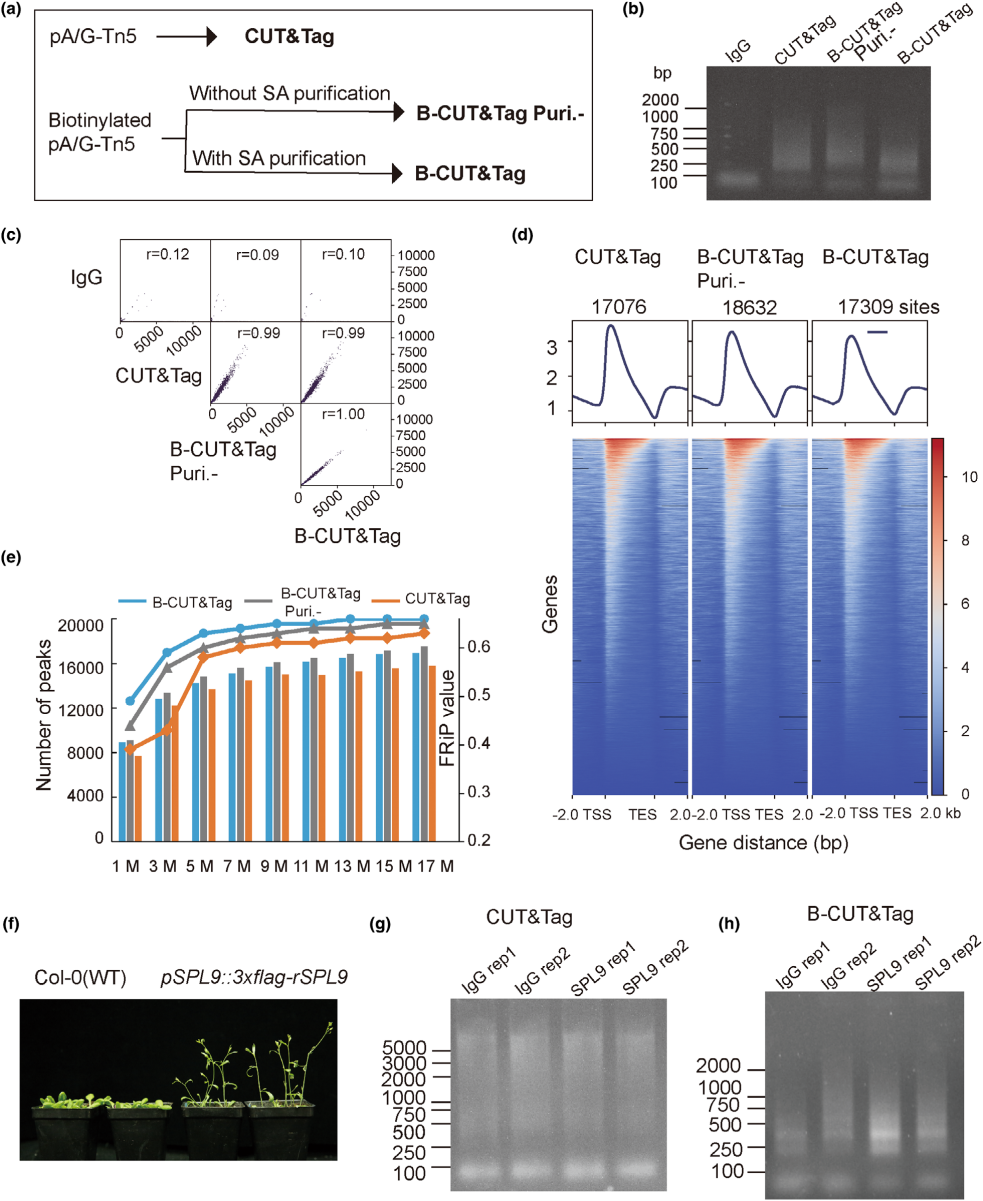
Effectiveness of B‐CUT&Tag compared with routine CUT&Tag under low input for histone modification and high‐input for TF‐DNA interaction. (a) Schematic diagram showed the differences in generating conventional CUT&Tag (CUT&Tag), B‐CUT&Tag without biotin‐SA purification (B‐CUT&Tag Puri.‐) and B‐CUT&Tag libraries. (b) Agarose gel electrophoresis analysis of IgG control, CUT&Tag, B‐CUT&Tag Puri.‐ and B‐CUT&Tag NGS libraries for histone H3K4me3 profiling with low input of nuclei (~1 μg chromatin). Normal mouse IgG (Millpore, 12–371) and anti‐H3K4me3 antibody (Abcam, ab8580) were used. (c) Pearson correlation analysis of H3K4me3 signal between IgG control, CUT&Tag, B‐CUT&Tag Puri.‐ and B‐CUT&Tag samples. (d) Heatmap analysis of H3K4me3 signals near protein‐coding genes. (e) Number of H3K4me3 peaks and fraction of reads in peaks (FRiP) as identified using different amounts of clean reads (in millions, M). (f) Early flowering phenotypes of *pSPL9::3xflag‐rSPL9* plants, which expressing 3xFLAG‐tagged miR‐156‐resistant form (*rSPL9*). Photographed 21 days after germination. (g and h) Agarose gel electrophoresis analysis of conventional CUT&Tag (g) and B‐CUT&Tag (h) NGS libraries for AtSPL9‐chromatin interactions with high‐input of nuclei (~5 μg chromatin/reaction).

We first determined whether B‐CUT&Tag could generate consistent chromatin landscapes in comparison to conventional CUT&Tag. CUT&Tag, B‐CUT&Tag without biotin‐SA magnetic bead purification (B‐CUT&Tag Puri.‐), and B‐CUT&Tag (B‐CUT&Tag) for H3K4me3 (a histone marker for active chromatin) were performed using low input of nuclei (equals ~1 μg chromatin for each reaction) isolated from shoots of 21‐day‐old wild‐type (Col‐0 background) *Arabidopsis* plants (Figure 2a). The bulk of the DNA fragments from above H3K4me3 CUT&Tag, B‐CUT&Tag Puri.‐ and B‐CUT&Tag libraries ranged from 250 to 750 base pairs (bp) after PCR amplification (Figure 2b). Analysis of H3K4me3 signals near protein‐coding genes in three different CUT&Tag samples showed high correlation and consistency (*r* ≥ 0.99) (Figure 2c). Similar numbers of peaks were called in CUT&Tag, B‐CUT&Tag Puri.‐ and B‐CUT&Tag samples (17 076, 18 632 and 17 309, respectively; Figure 2d). We summarized the number of H3K4me3 peaks and the fractions of reads in peaks (FRiP) obtained using different amounts of clean reads (in millions, M); FRiP values of up to ~0.6 were obtained using 9 m clean reads from three CUT&Tag samples and remained stable as the number of sampled clean reads was increased (Figure 2e). In summary, B‐CUT&Tag generates consistent profiles, including in terms of signal intensity, signal‐to‐noise ratio and number of peaks, compared with conventional CUT&Tag when low input of nuclei was applied for abundant H3K4me3 signals.

### B‐CUT&Tag efficiently profiles plant TF‐DNA interactions under high‐input of nuclei

To verify whether B‐CUT&Tag can be used to efficiently identify target genes of plant TFs, we conducted genome‐wide profiling of the targets of Squamosa promoter‐binding‐like protein 9 (SPL9) in *Arabidopsis*. For this, we used *pSPL9::3xflag‐rSPL9* plants (Wang *et al*., 2009) in which the 3xFLAG‐tagged miR‐156‐resistant form (*rSPL9*) was expressed from its native promoter. Relative to wild type (WT, Columbia‐0), *pSPL9::3xflag‐rSPL9* plants exhibited early flowering (Figure 2f). We performed B‐CUT&Tag using nuclei isolated from cross‐linked inflorescences of *pSPL9::3xflag‐rSPL9* plants, in which *AtSPL9* is highly expressed (Yu *et al*., 2010). B‐CUT&Tag and routine CUT&Tag assays for AtSPL9 were performed in parallel using high‐input of nuclei (equals ~5 μg chromatin/reaction, refer to Methods part for details). In AtSPL9 B‐CUT&Tag, the intact chromatin not tagmentated by pA‐Tn5 that unbound to SA magnetic beads were removed (Figure S2). The purified biotinylated target fragments were subjected to on‐beads PCR for the library construction, the bulk of the DNA fragments from the AtSPL9 B‐CUT&Tag libraries ranged from 250 to 750 base pairs (bp) after 18 cycles of PCR (Figure 2h), which is the regular product sizes for CUT&Tag assay. In comparison, without biotin‐SA based purification in conventional CUT&Tag for AtSPL9, the large amount of intact chromatin DNA in the PCR reaction affected the PCR efficiency, the resulting DNA fragments from the AtSPL9 CUT&Tag libraries showed a long smear from 250 bp up to more than 5000 bp after 18 cycles of PCR (Figure 2g) and failed to pass the Quality Control (QC) for NGS. These results indicated that B‐CUT&Tag efficiently profiles plant TF‐DNA interactions once high‐input of nuclei was used in the assay.

### Profiling of AtSPL9‐DNA interaction using B‐CUT&tag‐seq showed reproducible results when compared with ChIP‐seq

**Figure 3:**
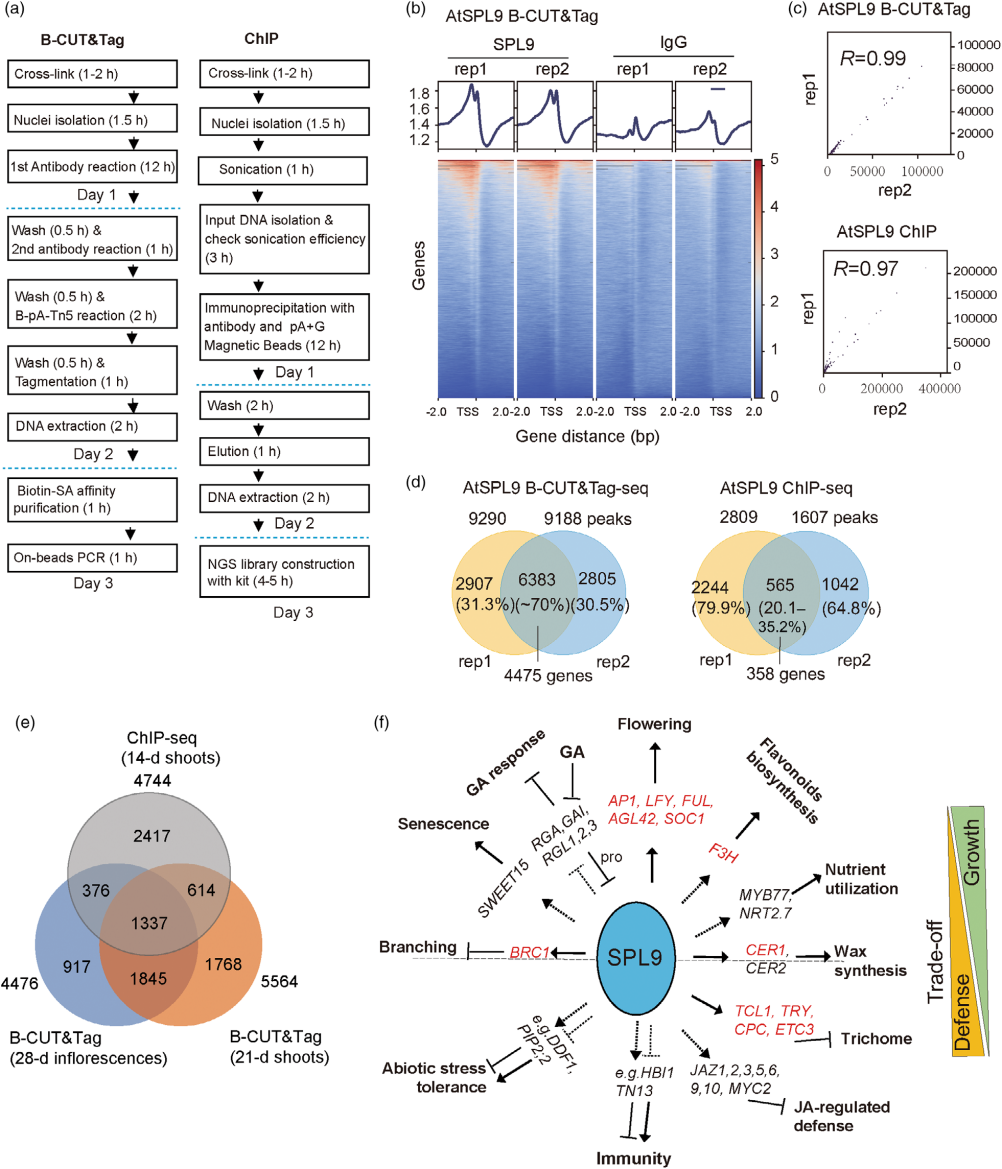
Successful application of B‐CUT&Tag to the identification of AtSPL9 target genes in *Arabidopsis*. (a) Workflow of B‐CUT&Tag vs. ChIP (b) Heatmap analysis of AtSPL9 B‐CUT&Tag‐seq signals near the TSSs of protein‐coding genes. (c) Scatterplot correlation of signals of AtSPL9 B‐CUT&Tag‐seq replicates (rep1 and rep2) and AtSPL9 ChIP‐seq replicates (rep1 and rep2), respectively, using Pearson's *r*. (d) Number of overlapped peaks and peak‐related genes for AtSPL9 B‐CUT&Tag‐seq and AtSPL9 ChIP‐seq when using same amount of nuclei (equals ~5 μg chromatin/reaction). (e) Interactive Venn graph showed overlapped potential target genes from ChIP using shoots of 14‐day‐old seedlings and B‐CUT&Tag using both inflorescences from 28‐day‐old plants and shoots of 21‐day‐old plants. (f) Functions of AtSPL9 in multiple biological processes. Well‐documented AtSPL9 targets represented in the B‐CUT&Tag results are indicated by red font and a solid arrow or inhibitory arrow for activation or inhibition of transcription; putative targets identified from B‐CUT&Tag and mRNA‐seq are indicated by a dotted arrow or inhibitory arrow.

We compared the procedures for the three‐day protocols for both B‐CUT&Tag and ChIP, B‐CUT&Tag outperforms ChIP as it is less device‐ and kit‐ dependent, it does not need expensive sonication device or the NGS library construction kit (Figure 3a). AtSPL9 B‐CUT&Tag‐seq signals were mainly distributed upstream of transcription start sites (TSSs), that is in promoter regions (Figure 3b). Two technical replicates showed high correlation (Figure 3c, Pearson's *r* = 0.99), with calling of 9290 and 9188 peaks, respectively (fold enrichment >2, *q*‐value <0.05). Of those, 6383 peaks (~70%) were overlapping and could be assigned to 4476 protein‐coding genes in the *Arabidopsis* genome (Figure 3d). We also performed AtSPL9 B‐CUT&Tag using nuclei isolated from shoots of 21‐day‐old *pSPL9::3xflag‐rSPL9* plants. In total, 3183 peaks (60%–70%) identified in that assay were shared with inflorescence samples (Figure 3e). In parallel of the AtSPL9 B‐CUT&Tag‐seq, we performed AtSPL9 ChIP‐seq using the same amount of nuclei (equals ~5 μg chromatin/reaction) isolated from 1 g cross‐linked inflorescences of *pSPL9::3xflag‐rSPL9* plants, unfortunately, we failed to obtain adequate resultant DNA, lead to low‐quality NGS libraries with low number of peaks (Figures 3d, S3). Alternatively, we provided AtSPL9 ChIP‐seq data using shoots of 14‐day‐old *pSPL9::3xflag‐rSPL9* plants, which yielded 5841 peaks corresponding to 4744 protein‐coding genes (fold enrichment >2, *q*‐value <0.05, data from the Jia‐Wei Wang lab), among which 1713 (36.1%) and 1951 (41.1%) peak‐associated genes overlapped with the target genes revealed by AtSPL9 B‐CUT&Tag‐seq of the inflorescences and shoots of 21‐day‐old plants, respectively (Figure 3e). These data indicate spatial and temporal variation of AtSPL9 regulation during plant growth and development.

We further evaluated the efficiency and consistency of B‐CUT&Tag by checking for the presence of multiple well‐established AtSPL9 target genes. This analysis revealed that B‐CUT&Tag extensively covered the well‐documented target genes of AtSPL9 implicated in multiple developmental and physiological pathways, including genes encoding a helix‐turn‐helix TF (*LFY*) and MADS‐box proteins (*AP1*, *SOC1*, *FUL* and *AGL42*) in the juvenile‐to‐adult phase transition and flowering (Lian *et al*., 2021; Smaczniak *et al*., 2012; Wang *et al*., 2009), MYB or MYB‐like proteins (*TCL1*, *TRY*, *CPC* and *ETC3*) in abaxial trichome initiation (Yu *et al*., 2010), flavanone 3‐hydroxylase (*F3H*) in flavonoid biosynthesis (Gou *et al*., 2011), a TCP family TF (*BRC1*) in shoot branching (Xie *et al*., 2020) and an alkane‐forming enzyme (*CER1*) in epidermal wax synthesis (Li *et al*., 2019) (Figure 3f). Gene ontology (GO) enrichment analysis further revealed that the potential target genes of AtSPL9 were significantly enriched (fold enrichment >2, *P* value <0.001) in multiple biological processes including meristem initiation, shoot and root development, lipid localization, abiotic/phytohormone stimulus responses and shade avoidance (Figure S4).

### Transcriptome analysis together with B‐CUT&Tag profiling identifies multiple potential AtSPL9 target genes involved in plant growth and defence balance

**Figure 4:**
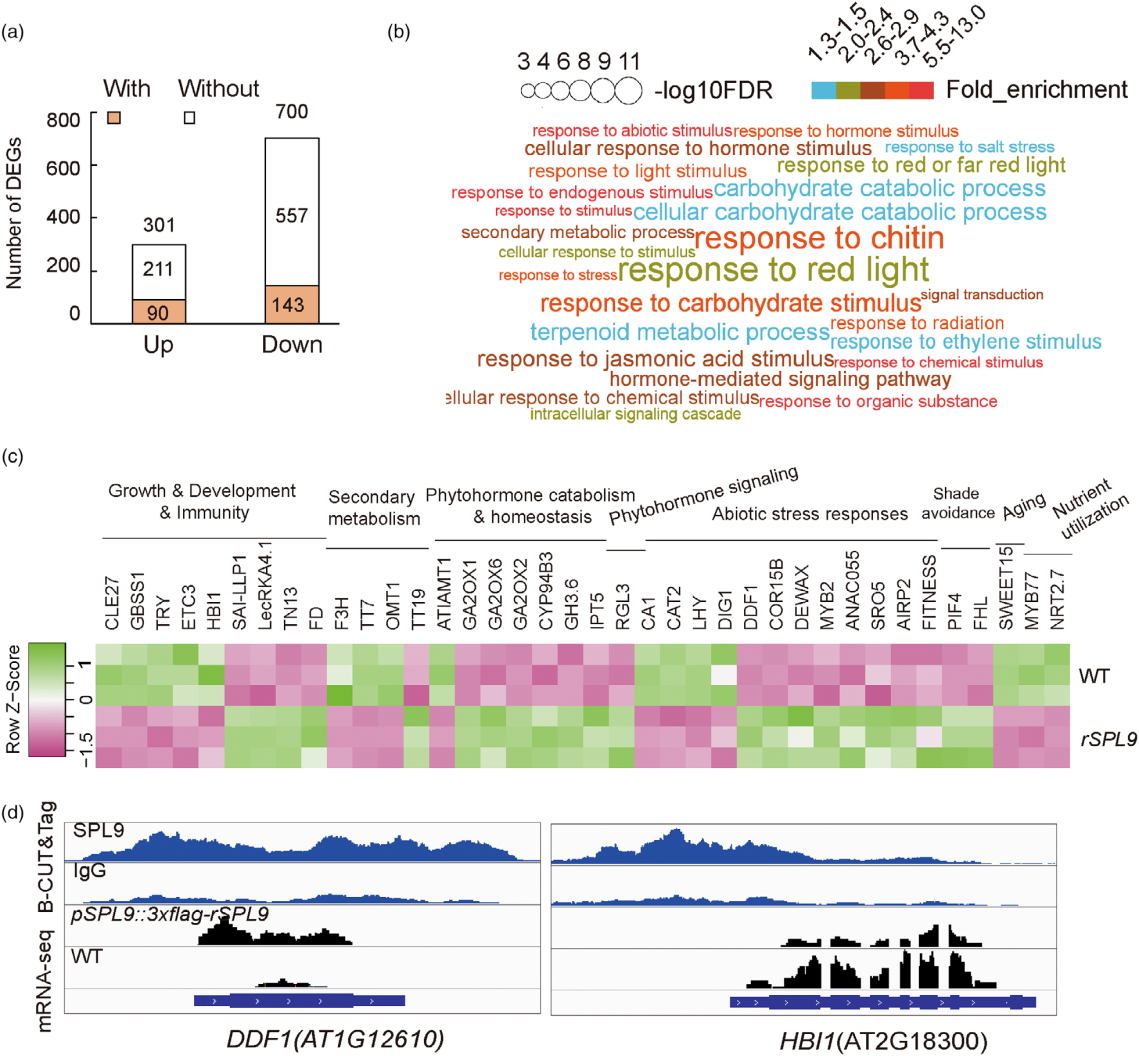
Transcriptome analysis together with B‐CUT&Tag profiling highlight multiple potential target genes of AtSPL9 involved in plant growth and defence balance. (a) Number of DEGs identified in *pSPL9::3xflag‐rSPL9* plants compared with WT and their overlap with B‐CUT&Tag peak‐related genes. With: DEGs with B‐CUT&Tag peaks; Without: DEGs without B‐CUT&Tag peaks. (b) GO terms enriched among DEGs having B‐CUT&Tag peaks. (c) Heatmap of selected DEGs with B‐CUT&Tag peaks that are involved in multiple biological processes. (d) Representative DEGs with B‐CUT&Tag peaks that are involved in growth and defence.

mRNA‐seq was performed using inflorescences from WT and *pSPL9::3xflag‐rSPL9* plants and revealed 1001 differentially expressed genes (DEGs, fold change >1.5 or <0.67; FDR <0.05). Among them, 233 genes (90 up‐regulated and 143 down‐regulated) overlapped with the list of prospective target genes from B‐CUT&Tag (Figure 4a, Table S3). GO enrichment analysis of the AtSPL9‐targeted DEGs revealed them to be significantly enriched in responses to abiotic stimulus or phytohormones (fold enrichment >2, FDR < 0.05) (Figure 4b), indicating that AtSPL9 regulates pathways partially shared with the response pathways of multiple stimuli.

We are interested in the DEGs also represented in B‐CUT&Tag peaks, which are most likely target genes of AtSPL9. Most of them constitute new discoveries as potential targets of AtSPL9, and these genes are broadly involved in growth, development and immunity, secondary metabolism, phytohormone catabolism & homeostasis, phytohormone signalling, abiotic stress responses, shade avoidance, aging and nutrient utilization (Figure 4c, Table S4). It has been reported that DELLA proteins, the key signalling factors in the GA pathway, inhibit AtSPL9 activity through direct interaction at the protein level (Mao *et al*., 2017). Interestingly, the B‐CUT&Tag results indicated that AtSPL9 binds to the promoter regions of DELLA genes (*RGA*, *GAI* and *RGL1*, *2* and *3*) (Figure 3f), and *RGL3* was found to be down‐regulated in the inflorescences of *pSPL9::3xflag‐rSPL9* plants (Table S4), implying possible feedback between AtSPL9 and DELLA in GA responses (Figure 3f). Figure 4c and Table S4 summarize ten DEGs having mutant or over‐expression lines with documented phenotypes under salt, cold, or drought stress. For example, the salinity‐responsive DWARF AND DELAYED FLOWERING 1 (*DDF1*) gene was down‐regulated in *pSPL9::3xflag‐rSPL9* plants (Figure 4c,d); plants overexpressing this gene exhibit delayed flowering and dwarfism, reduction of GA biosynthesis and increased tolerance to high levels of salt (Magome *et al*., 2008). Combining the expression profiles of multiple abiotic stress response‐related DEGs (including *DDF1*, *SRO5*, *ANAC055*, *AIRP2*, *MYB2* and *COR15B*) with their documented phenotypes (Abe *et al*., 2003; Borsani *et al*., 2005; Cho *et al*., 2011; Fu *et al*., 2018; Magome *et al*., 2008; Thalhammer and Hincha, 2014) (Table S4) suggests *pSPL9::3xflag‐rSPL9* plants to be more sensitive to abiotic stresses, which is consistent with previous reported phenotypes for *rSPL9* plants under salt and osmotic stress (Cui *et al*., 2014).

### B‐CUT&Tag‐qPCR for validation of histone modification levels at specific gene loci

**Figure 5:**
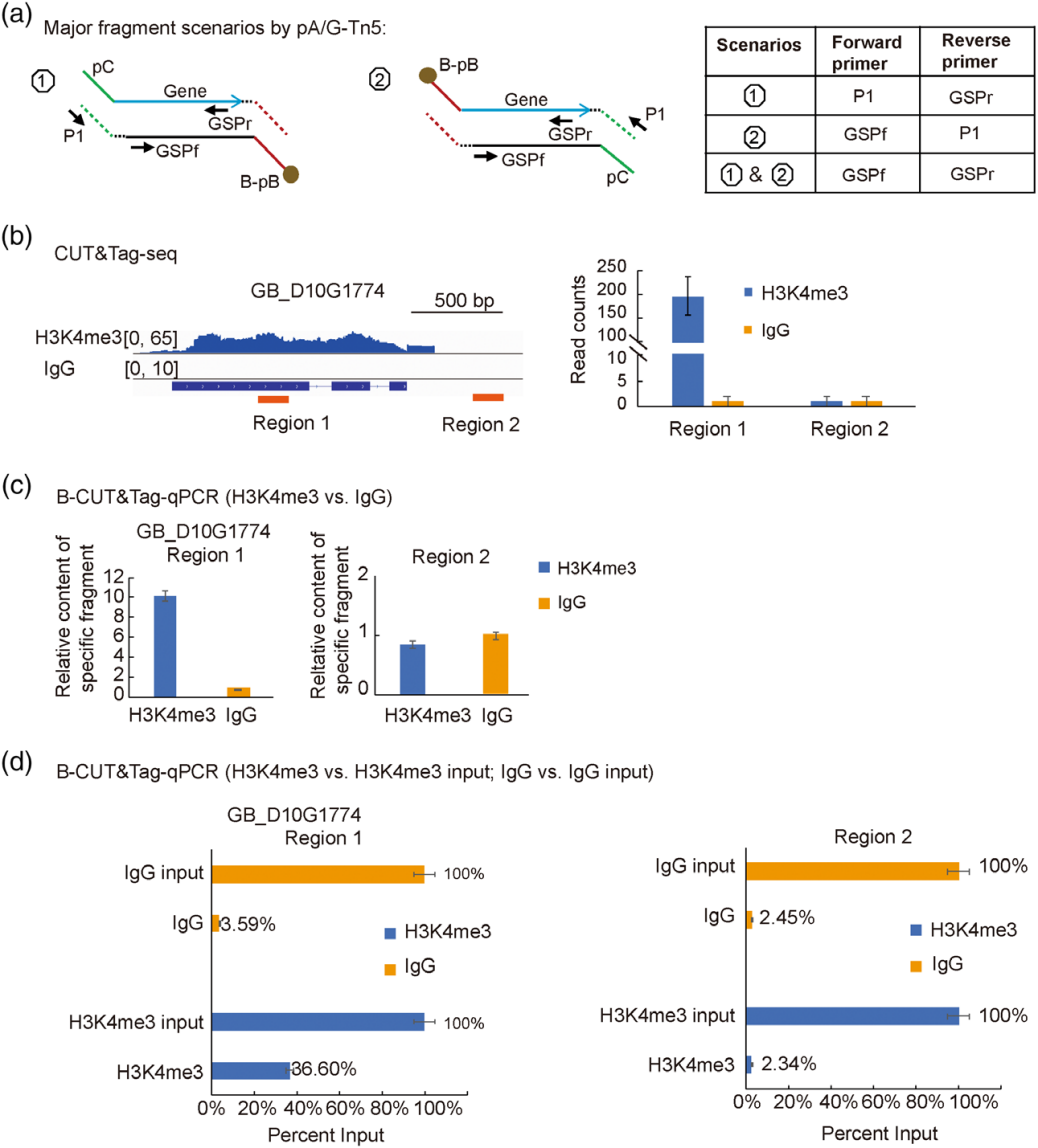
B‐CUT&Tag‐qPCR validates H3K4me3 profiles for known chromatin sites in the cotton genome. (a) Schematic diagram showing the major tagmentation scenarios generated by pA/G‐Tn5 transposase homo‐dimers. The primers for qPCR identification of each scenario are provided. (b) CUT&Tag‐seq results showed the H3K4me3 modification status at *GB_D10G1774* gene locus. IGV profiles depict the regions with high and low levels of H3K4me3 modification (Region 1 and Region 2, respectively) that are selected for validation in qPCR. Read counts that uniquely mapped to Region 1 and Region 2 are summarized. (c) Normalized qPCR results show the relative content of fragments from Region 1 and Region 2 in the H3K4me3 assay group compared with the IgG control group. GSPf/GSPr for *GB_D10G1774* were used in the qPCR. (d) Normalized qPCR results showing the percent input of specific fragments. H3K4me3 input or IgG input represent the DNA from H3K4me3 or IgG B‐CUT&Tag sample before subjecting it to biotin‐SA‐based purification. H3K4me3 or IgG represent DNA samples after biotin‐SA‐based purification. GSPf/GSPr for *GB_D10G1774* were used in the qPCR.

To determine whether B‐CUT&Tag could likewise be combined with qPCR for validation of the known binding sites, we established the B‐CUT&Tag‐qPCR procedure by validating H3K4me3 profiles for representative sites in the cotton genome. We take advantage of the previous B‐CUT&Tag‐seq data for H3K4me3 modification in cotton plants (Tao *et al*., 2020) and checked the H3K4me3 levels at specific gene loci as a reference for comparison. We first test the primer pairs that can be used in the qPCR for B‐CUT&Tag samples. Theoretically, there was three different assembly ways of pA‐Tn5 transposome and resulted as four scenarios for adaptor ‘pasting’ after Tn5 tagmentation (Figure S5a). To roughly estimate the proportion of different tagmentation scenarios, we performed Tn5 tagmentation of genomic DNA followed with qPCR to identify the relative content of products from different scenarios, and results showed that the scenario 1 and scenario 2 products accounted for ~99.97% of the tagmentation (Figures 5a, S5b), suggesting that the pA‐Tn5 transposome homo‐dimer harbours both adaptor AB and adaptor AC is the major form after transposome assembly. As an example, we check the H3K4me3 modification levels at *GB_D10G1774* gene locus. The fragments from scenario 1 and scenario 2 were quantified using the primer pairs P1/GSPr_GB_D10G1774 and P1/GSPf_GB_D10G1774, respectively (Figure 5a), in which primer P1 harbours the 15 bp 5′ adaptor sequence for Illumina sequencing from adaptor Primer C but not the Tn5 mosaic end (ME) sequence (refer to Table S1 for details), while GSPf_GB_D10G1774 and GSPr_GB_D10G1774 are gene‐specific forward (GSPf) and reverse primers (GSPr) for *GB_D10G1774* (Table S1). Unfortunately, we found non‐specific amplification by the single P1 primer, this is more likely resulted from the annealing and extension of single‐strand DNAs (ssDNAs) from the same target site recovered from scenario 1 and scenario 2, respectively (Figure S6a,b). Adding an on‐beads extension step using DNA polymerase before DNA retrieval step significantly reduce non‐specific priming by primer P1 (ΔCt = 5.25) (Figure S6c,d). However, considering the background non‐specific priming by primer P1, qPCR using the primer pairs of P1/GSPf or P1/GSPr is not applied.

We then evaluated gene‐specific primer pairs (GSPf/GSPr) in the qPCR reaction. For the gene *GB_D10G1774*, CUT&Tag‐seq results indicated that H3K4me3 signals were enriched in the gene body and weak in the 3′ downstream region (Figure 5b). B‐CUT&Tag‐qPCR targeted to both the high signal region (Region 1) and the low signal region (Region 2) was performed, consistently, results indicated that the target fragments covering Region 1 having 10.2‐fold enrichment in the H3K4me3 assay group normalized to IgG control, while the target fragments covering Region 2 showed no significant enrichment after normalization (Figure 5c).

In an alternative normalization method, we used each DNA sample from before biotin‐SA‐based purification as the reference for normalization of qPCR data. With this normalization, 36.60% and 3.59% of respective fragments in the H3K4me3 assay group and IgG control group covered Region 1 after biotin‐SA‐based purification, while 2.45% and 2.34% of fragments covered Region 2 (Figure 5d). Consistent results was also observed at other gene loci, including *GB_D03G0642* and *GB_A11G13949*, as established by qPCR using gene‐specific primer pairs (Figure S7). Thus, B‐CUT&Tag‐qPCR using target‐specific primer pairs is applicable for validation of histone modifications at specific gene loci, and the cycle threshold (Ct) values from both an IgG control and input DNA can be used for normalization.

### B‐CUT&Tag‐qPCR for profiling the binding of a TF

**Figure 6:**
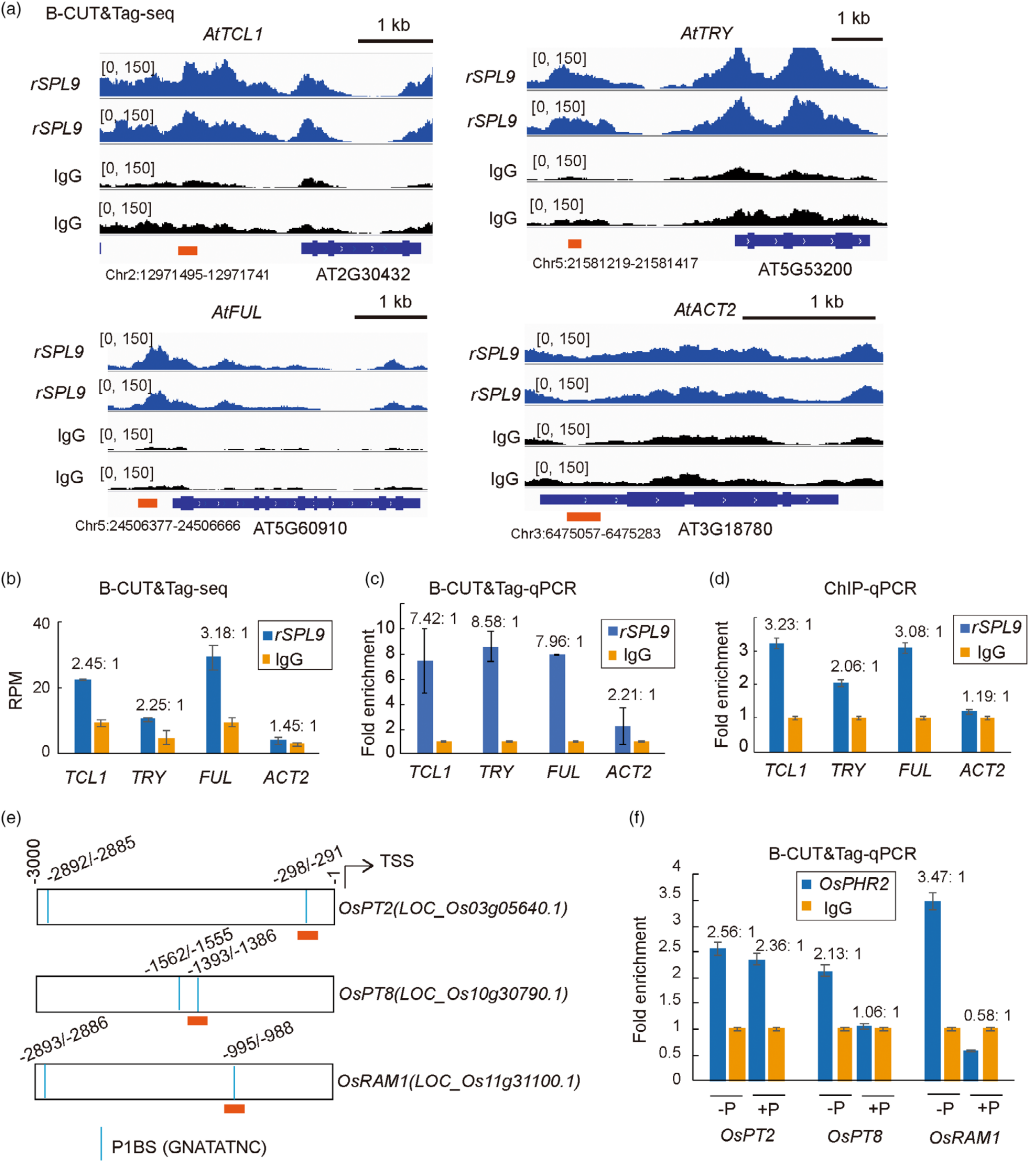
B‐CUT&Tag‐qPCR validates TF‐DNA binding for known target sites in *Arabidopsis* and rice. (a) IGV profiles depicting the B‐CUT&Tag‐seq binding profiles of AtSPL9 at well‐established target gene loci including *AtTCL1*, *AtTRY* and *AtFUL*. The *AtACT2* gene locus was used as negative control. (b) Reads counts per million mapped reads (RPM) that uniquely mapped to the indicated promoter regions (orange boxes from panel a). (c) Normalized qPCR results showing the relative enrichment of fragments from indicated promoter regions of genes (orange boxes from panel a) in the rSPL9 assay group (anti‐FLAG antibody) compared with the IgG control group. Data were summarized from two independent biological replicates of B‐CUT&Tag‐qPCR. The binding of AtSPL9 to the indicated promoter regions (orange boxes) of *AtTCL1*, *AtTRY*, *AtFUL* were previously confirmed by ChIP‐qPCR (Wang *et al*., 2009; Yu *et al*., 2010), the same gene‐specific primer pairs were used. (d) Normalized ChIP‐qPCR results showing the relative enrichment of fragments from indicated promoter regions of genes (orange boxes from panel a) in the rSPL9 assay group (anti‐FLAG antibody) compared with the IgG control group. (e) Schematic diagram of P1BS (GNATATNC) sites in the promoter regions of rice *OsPT2*, *OsPT8* and *OsRAM1* genes. (f) Normalized qPCR results showing relative enrichment of fragments from the indicated P1BS sites (orange boxes from panel e) in the OsPHR2 assay group (anti‐FLAG antibody) compared with the IgG control group under phosphate deficient (−P) or sufficient (+P) conditions.

To determine whether the B‐CUT&Tag‐qPCR procedure we established for assessing histone modification levels was applicable to validating the occupancy of TFs, we employed this method to determine the binding of AtSPL9. Three well‐established target genes of AtSPL9 were selected: *TCL1* and *TRY*, involved in trichome development (Yu *et al*., 2010), and *FUL*, involved in flowering control (Wang *et al*., 2009). The gene‐specific primer pairs used for B‐CUT&Tag‐qPCR were the same with previously documented for ChIP‐qPCR assays (Wang *et al*., 2009; Yu *et al*., 2010). The *ACT2* gene was used as control. In parallel, we summarized the read counts from B‐CUT&Tag‐seq data that mapped to the qPCR target region (Figure 6a). The ratios of read counts per million mapped reads (RPM) for the qPCR target regions of *TCL1*, *TRY*, *FUL* and *ACT2* were 2.45, 2.25, 3.18 and 1.45, respectively, in the rSPL9 assay group (anti‐FLAG antibody) compared with the IgG control (Figure 6b). Consistent with these data, B‐CUT&Tag‐qPCR results showed 7.42‐, 8.58‐, 7.96‐ and 2.21‐fold enrichment for the indicated regions of *TCL1*, *TRY*, *FUL* and *ACT2* in the rSPL9 assay group relative to the IgG control (Figure 6c). In comparison, a parallel ChIP‐qPCR results showed 3.23‐, 2.06‐, 3.08‐ and 1.19‐fold enrichment for the indicated regions of *TCL1*, *TRY*, *FUL* and *ACT2* in the rSPL9 assay group relative to the IgG control (Figure 6d). These results indicated that B‐CUT&Tag‐qPCR had comparable sensitivity with ChIP‐qPCR.

We further applied B‐CUT&Tag‐qPCR to determine the binding of OsPHR2, an important TF that functions in phosphate homeostasis in rice. Three known target genes were selected for analysis: the low‐affinity phosphate (Pi) transporter *OsPT2* (Liu *et al*., 2010), which is the direct target of *OsPHR2* and responsible for most of the *OsPHR2*‐mediated accumulation of excess shoot Pi; the high‐affinity Pi transporter *OsPT8*, which is also critical for Pi homeostasis (Jia *et al*., 2011); and the arbuscular mycorrhizae symbiosis‐associated marker gene *OsRAM1*, which is regulated by an *OsPHR2*‐centered network (Shi *et al*., 2021). The B‐CUT&Tag‐qPCR assay was performed using nuclei isolated from cross‐linked shoots of *OsPHR2‐Ov1* rice plants (an transgenic plant overexpressing *OsPHR2* with FLAG tagging) (Kong *et al*., 2021). We identified the PHR1 binding site (P1BS; GNATATNC) (Rubio *et al*., 2001) in the promoters of *OsPT2, OsPT8* and *OsRAM1* and designed qPCR primers with target regions covering the indicated P1BS *cis*‐element (Figure 6e). qPCR results showed that, under the Pi‐sufficient (+P) condition, there was no enrichment of target regions for *OsPT8* and *OsRAM1* in the *OsPHR2* assay group (anti‐FLAG antibody) relative to the IgG control group; in contrast, 2.36‐fold enrichment of the target region was observed for *OsPT2*, which is consistent with the expression pattern of *OsPT2* in *OsPHR2*‐overexpressing rice plants (Liu *et al*., 2010). Meanwhile, under a Pi‐deficient (−P) condition, 2.56‐, 2.13‐ and 3.47‐fold enrichment was, respectively, observed for the target regions of *OsPT2*, *OsPT8* and *OsRAM1* in the *OsPHR2* assay group compared with IgG control (Figure 6f). These results indicate different regulation effects of *OsPHR2* on Pi homeostasis and mycorrhizal symbiosis under Pi‐sufficient (+P) and Pi‐deficient (−P) conditions.

### General strategy of B‐CUT&tag for plant TFs

**Figure 7:**
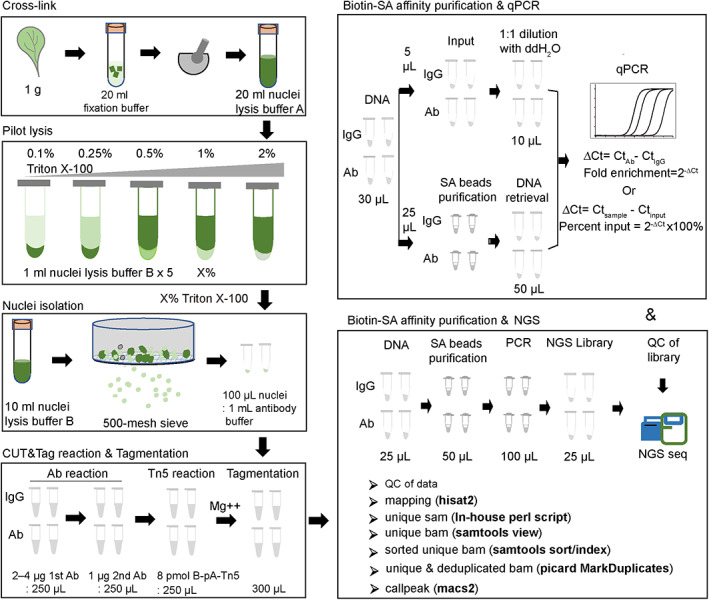
Strategy of plant B‐CUT&Tag for profiling TF‐chromatin interactions.

To summarize, Figure 7 illustrates the comprehensive methodological strategy for B‐CUT&Tag in plants (refer to Methods part for details). Firstly, for cross‐link, the plant tissues were fixed with 1% formaldehyde and quenched with 0.2 m glycine. Secondly, for nuclei isolation, a pilot lysis using a series of Triton X‐100 concentrations is recommended, with 100 mg liquid nitrogen grinded tissue in each 2 mL nuclei lysis buffer. Taking leaf tissue as an example, successful lysis is indicated by accumulation of white/grey nuclei at the bottom and a dark green supernatant after centrifuging at low speed (<500 ***g*** for 4 min). The lysate is then filtered through a 500‐mesh stainless steel sieve to collect the nuclei. Thirdly, the B‐CUT&Tag reaction is performed in an optimized volume with respect to the amount of nuclei, antibody, pA/G‐Tn5. Finally, we demonstrated the biotin‐SA magnetic beads‐based DNA purification and the subsequent on‐beads PCR to be feasible for NGS library construction (B‐CUT&Tag‐seq), along with an optimized qPCR procedure for confirming TF‐DNA binding (B‐CUT&Tag‐qPCR).

## Discussion

### B‐CUT&Tag provided new clues for the role of AtSPL9 in modulating growth‐defence trade‐offs

Beyond the simple affinity of a TF for its corresponding 6–12 bp‐long *cis*‐regulatory DNA sequence, TF binding and its impact on gene expression are influenced by many different mechanisms, including chromatin accessibility (nucleosome positioning), cofactor proteins, DNA architecture and more (Spitz and Furlong, 2012). Comparisons of TF occupancy from ChIP‐seq data and differentially expressed genes (DEGs) from mRNA‐seq in yeast (Ucar *et al*., 2009), *Drosophila melanogaster* (Jakobsen *et al*., 2007) and mammalian systems (Vokes *et al*., 2008) have revealed a relatively small overlap between TF binding and gene expression. In our study, B‐CUT&Tag‐seq data revealed totally 4475 potential AtSPL9‐target genes, while RNA‐seq revealed totally 1001 DEGs, in which 233 genes are overlapping, accounting for 23% (233/1001) of the DEGs. This overlap rate is comparable to previous values reported in mammalian systems and plants (10–25%) (Dong *et al*., 2019; Vokes *et al*., 2008; Zhang *et al*., 2017). AtSPL9 has been revealed to positively regulate gene expression by directly binding to a promoter (Wang *et al*., 2009) and to negatively regulate gene expression (e.g. of anthocyanin biosynthesis genes *F3’H*, *DFR*, *ANS* and *UGT78D2*) through indirectly destabilizing the MYB‐bHLH‐WD40 activator complex (Gou *et al*., 2011). We identified 90 up‐regulated DEGs and 143 down‐regulated DEGs as also having B‐CUT&Tag peaks (Figure 5a), which were significantly enriched for the terms response to abiotic stimulus and response to phytohormone stimulus (Figure 5b). Although further experimental confirmation of these TF‐gene interactions remains needed, our data provide new clues for a role of AtSPL9 in coordinating the growth‐defence trade‐offs in plants. Briefly, plants with high AtSPL9 activity adjust their growth/development and exhibit early flowering and accelerated senescence. In this way, plants prioritize responses that improve their capacity to complete their life cycle quickly (i.e. stress escape) while becoming more susceptible to abiotic stress (Cui *et al*., 2014; Gou *et al*., 2011), insect herbivory (Mao *et al*., 2017) or pathogens.

### B‐CUT&Tag‐seq and B‐CUT&Tag‐qPCR provide general strategies for profiling plant TF occupancy

We have developed B‐CUT&Tag‐seq and B‐CUT&Tag‐qPCR procedures that are likely to be widely applied in profiling plant TF‐DNA interactions. CUT&Tag provides outstanding performance with low chromatin input even at the single‐cell level (Bartosovic *et al*., 2021; Ouyang *et al*., 2021); however, for most scenarios involving plant tissues such as leaves, stems, roots, inflorescences, fruits and seeds, the amount of input sample does not appear to be the limiting factor. We have employed CUT&Tag assays in plants for years and identified the most likely limiting factors for its successful application as the isolation of intact nuclei and the abundance of target TFs. For the isolation of intact nuclei, we provided a detailed methodology in which a pilot assay that determining the appropriate concentration of Triton X‐100 was introduced, and also a 500‐mesh sieve was used to filter the nuclei after lysis (Figure 7, refer to method part for details). Our B‐CUT&Tag procedure was designed for profiling the occupancy of plant TFs using quantities of input nuclei equivalent to or less than those needed for ChIP; we further combined biotinylated Tn5 transposase‐mediated tagmentation with biotin‐SA‐based purification, thus optimizing the purification of DNA fragments that specifically resulting from Tn5 tagmentation. As a result, large amounts of intact chromatin not targeted by specific proteins were excluded (Figure S2), which minimized the negative effects of such chromatin in downstream NGS library construction and sequencing. In principle, solid phase reversible immobilization (SPRI) methodology‐based DNA size selection by purification beads (e.g. AMPure XP) might be applicable to remove larger intact chromatin DNA and recover desired size DNA fragments after traditional CUT&Tag. However, the efficiency, sensitivity and specificity between SPRI‐based selection and biotin‐SA based purification in B‐ CUT&Tag need to be compared in further experiment. Our results indicated that biotin‐SA based purification methodology was easy to perform and efficiently purified target DNA for the following NGS library construction (Figure 2h). Thus, our B‐CUT&Tag procedure is recommended when traditional CUT&Tag procedure failed to obtain qualified libraries, which might be especially suitable for TFs with lower abundance or with lower number of target sites that need higher input of nuclei in the reaction. Importantly, for the TFs that with a relatively lower abundance or lower number of target sites, multiple technical replicates of reactions are recommended; the resultant DNA from multiple technical replicates can be combined to undergo biotin‐SA‐based purification. Thus, B‐CUT&Tag enables flexibility in the amount of input nuclei depending on the abundance of target TFs. Notably, B‐CUT&Tag still outperformed traditional ChIP in terms of its low dependence on devices/kits (Figure 3a) and high consistency between biological replicates (Figure 3c). Our B‐CUT&Tag‐qPCR procedure is applicable for profiling binding of TFs in both monocot (rice) and dicot (cotton and *Arabisopsis*) plants for the known target sites.

### Prospects of B‐CUT&Tag in *cis*‐regulatory element annotation for crop improvement

Since its invention, CUT&Tag has provided an alternative method for profiling protein‐DNA interactions in both animals and plants. CUT&Tag for bulk histone modification has been successfully applied to investigate the epigenomic landscapes of monocot rice and dicot rapeseed and cotton (Ouyang *et al*., 2021; Tao *et al*., 2020). In addition to the bulk histone modification states that reflect chromatin accessibility, annotation of biologically significant *cis*‐regulatory elements relating to yield, quality and stress tolerance is of great importance in crop production. We provide for the first time high‐quality plant TF occupancy profile data obtained using CUT&Tag‐seq and also establish a CUT&Tag‐qPCR procedure with which to examine binding of TFs. Our results suggest that this CUT&Tag strategy can be widely applied in plants. Moreover, as it is very difficult and costly to systematically produce reliable antibodies for the specific recognition of individual plant proteins (Lu *et al*., 2020), when generating the overexpressing transgenic plants, we fused the open reading frame (ORF) of the TF with FLAG tag (DYKDDDDK) and utilized a commercial anti‐FLAG antibody to recognize the target tag‐fused protein. Thus, this B‐CUT&Tag method using an antibody against specific protein tag has great promise for applications examining the binding of chromatin proteins and annotating *cis*‐regulatory TF‐binding elements for crop improvement.

## Methods

### Plant materials and growth conditions

*Arabidopsis* wild type (WT) and *pSPL9::3xflag‐rSPL9* lines were grown in a growth chamber at 22 °C with a 16‐h light/8‐h dark photoperiod. The 21‐day‐old *pSPL9::3xflag‐rSPL9* plants or inflorescences from 28‐day‐old *pSPL9::3xflag‐rSPL9* plants were used in B‐CUT&Tag. Upland cotton (*Gossypium hirsutum*) was grown in a growth chamber at 28 °C with a 16‐h light/8‐h dark photoperiod. Leaves from 30‐day‐old seedlings were used in cross‐linking CUT&Tag (X‐CUT&Tag). Rice (*Oryza sativa* L. cv. Nipponbare) *OsPHR2‐Ov1* plants (a transgenic plant overexpressing *OsPHR2* with FLAG tagging) (Kong *et al*., 2021) were grown in a growth chamber with a 14‐h light (30 °C)/8‐h dark (25 °C) photoperiod and thermoperiod. Seven‐day‐old rice seedlings initially grown on Pi‐sufficient medium were subjected to P‐sufficient (+P, 200 μm KH_2_PO_4_) and P‐deficient (−P, 0 μm KH_2_PO_4_) conditions. In the Pi‐deficient medium, phosphate buffer was replaced by equimolar amounts of KCl (Kong *et al*., 2021). Three days later, rice shoots were harvested for B‐CUT&Tag.

### CUT&Tag

Routine CUT&Tag for profiling histone H3K4me3 or H3K27me3 was performed as described (Tao *et al*., 2020). Due to the abundance of histone H3K4me3 and H3K27me3 modification, small input of nuclei (equals ~1 μg chromatin) was used in each reaction, and only the primary antibodies including normal mouse IgG (12–371; Millpore, Burlington, MA, USA) for control, anti‐H3K4me3 antibody (ab8580; Abcam, Cambridge, Cambridgeshire, UK) and anti‐ H3K27me3 antibody (ab6002; Abcam) were used.

### B‐CUT&Tag‐seq & B‐CUT&Tag‐qPCR

The B‐CUT&Tag protocol was developed from a previous method with modifications (Tao *et al*., 2020). All buffers used were the same as described (Tao *et al*., 2020). Sequences of primers A, B and C for generating adaptor AB and adaptor AC are provided in Table S1. Primer B was 5′‐biotin‐TEG modified. Primer A, B and C was diluted in annealing buffer (10 mm Tris pH 8.0, 50 mm NaCl, 1 mm EDTA) to make a concentration of 100 μm. In two PCR tubes, set up the following reactions by mixing 10 μL primer A with 10 μL primer B (tube 1 for adaptor AB); and 10 μL primer A with 10 μL primers C (tube 2 for adaptor AC). The adaptors were generated using the program in the PCR machine (heat lid, 75°C for 15 min, 60 °C for 10 min, 50 °C for 10 min, 40 °C for 10 min, 25 °C for 30 min). The adaptor AB and adaptor AC were mixed at 1:1 ratio, designated as ‘adaptor mix’ (50 μm). Five microlitre of commercially available pA‐Tn5 (7.5 pmol/μL) for example Vazyme, or Abclonal was complexed with 0.875 μL adaptor mix and 3.5 μL of coupling buffer at 37°C for 60 min to assemble the Tn5 transposase.

For B‐CUT&Tag for TF, cross‐linking was first performed, in which each 1 gram of tissue was fixed using 20 mL fixation buffer (10 mm Tris pH 8.0, 10 mm KCl, 0.5 mm spermidine, 1 mm EDTA, β‐Mercaptoethanol, 1% formaldehyde) for 10 min under vacuum; the cross‐linking was subsequently stopped by adding 2 mL of 2 m glycine. The cross‐linked tissue were washed using 50 mL ddH_2_O for three times. After washing, the tissue were transferred to paper towels to absorb excess water.

For nuclei isolation, (optional) a pilot assay was performed to determine the appropriate concentration of Triton X‐100 in the lysis buffer: Half a gram cross‐linked tissue was ground into fine power in liquid nitrogen, then transferred to 50 mL falcon tubes. Ten milliliter of ice‐cold nuclei lysis buffer A (10 mm Tris pH 8.0, 10 mm KCl, 0.5 mm spermidine, 1 mm EDTA, β‐Mercaptoethanol) was added and the material resuspended evenly. The suspension (~10 mL) was transferred to five 2‐mL tubes followed by centrifugation at 400 ***g*** at 4°C for 5 min. The supernatant was then decanted and the pellet (~100 mg/tube) was resuspended by 1 mL nuclear isolation buffer B (10 mm Tris pH 8.0, 10 mm KCl, 0.5 mm spermidine, 1 mm EDTA, X% Triton X‐100, 0.1% protease inhibitor cocktail) with 0.1%, 0.25%, 0.5%, 1% and 2% Triton X‐100, respectively, for each tube. Take plant leaves as an example, the appropriate concentration of Triton X‐100 for lysis is indicated by accumulation of white/grey nuclei at the bottom and a dark green supernatant after centrifugation (400 ***g*** for 5 min). For cross‐linked inflorescences or shoots of *Arabidopsis* for AtSPL9 assay, and cross‐linked shoot of seven‐day‐old rice seedlings for OsPHR2 assay in this study, the appropriate concentration of Triton X‐100 is 0.5%.

One gram cross‐linked tissue was ground into fine power in liquid nitrogen, then transferred to 50 mL falcon tubes. Twenty milliliter of ice‐cold nuclei lysis buffer A was added and the material resuspended evenly, followed by centrifugation at 400 ***g*** at 4 °C for 5 min. The supernatant was then decanted and the pellet was resuspended by adding 10 mL nuclei lysis buffer B containing 0.5% Triton X‐100, followed by incubation on ice for 5–10 min. The resulting lysate (~10 mL) was transferred to five 2‐mL tubes and the pellet was collected by centrifugation for 4 min at 400 ***g***, 4 °C. The supernatant was then decanted and the pellet was resuspended by adding 10 mL nuclei wash buffer (10 mm Tris pH 8.0, 150 mm NaCl, 0.5 mm spermidine, 0.1% protease inhibitor cocktail). The nuclei suspension was filtered through an autoclaved 500‐mesh sieve to remove larger tissue debris. The nuclei were collected in 2‐mL tubes by centrifugation for 4 min at 400 ***g***, 4°C and all the nuclei was combined into a fresh 1.5 mL tube and washed three times using 1 mL nuclei wash buffer. We estimated nuclei yield based on the pellet volume produced by low‐speed centrifugation (400 ***g***). For one gram cross‐linked tissue, ~100 μL nuclei in volume was finally obtained.

In setting up for the antibody reaction, 100 μL nuclei in volume (equals ~20 μg chromatin) was resuspended in 1 mL antibody buffer (50 mm Tris pH 8.0, 1 mm EDTA, 150 mm NaCl, 0.5 mm spermidine, 1 mg/mL BSA, 0.1% protease inhibitor cocktail, 0.05% w/v digitonin). Each reaction consisted of 250 μL above nuclei suspension. We set up two replicates each for normal mouse IgG (12–371; Millpore) and anti‐FLAG antibody (ab125243; Abcam). Two microlitre (2 μg) antibody was added to 250 μL nuclei suspension and incubated with gentle rotation (12 rpm) at 4°C overnight. After the antibody reaction, the nuclei were collected by centrifugation for 4 min at 400 ***g***, 4°C and washed gently for 5 min using 800 μL IP wash buffer (10 mm Tris pH 8.0, 150 mm NaCl, 0.5 mm spermidine, 0.1% protease inhibitor cocktail, 0.05% v/v digitonin). One μg of rabbit anti‐mouse IgG H&L (HRP) (ab6728; Abcam) in 250 μL IP wash buffer was added to each reaction and incubated with gentle rotation (12 rpm) at room temperature for one hour. Afterwards, the nuclei were collected by centrifugation for 4 min at 400 ***g***, 4°C and washed three times using 800 μL IP wash buffer to remove the unbound secondary antibody.

For transposase incubation, 2 μL of biotinylated pA‐Tn5 (8 pmol, commercial source, e.g Vazyme or Abclonal) in 250 μL transposase incubation buffer (10 mm Tris pH 8.0, 300 mm NaCl, 0.5 mm spermidine, 0.1% protease inhibitor cocktail, 0.05% w/v digitonin) was added to each reaction and incubated with gentle rotation (12 rpm) at room temperature for 2 h. Subsequently, the products were the nuclei were collected by centrifugation for 4 min at 400 ***g***, 4 °C and washed three times using 800 μL IP wash buffer to remove unbound pA‐Tn5.

For tagmentation, 300 μL tagmentation buffer was added (10 mm Tris pH 8.0, 300 mm NaCl, 0.5 mm spermidine, 0.1% protease inhibitor cocktail, 10 mm MgCl_2_, 0.05% w/v digitonin) and incubated at 37 °C for one hour.

For DNA extraction, 30 μL 10% SDS and 10 μL 0.5 m EDTA were added to stop the tagmentation reaction, and the tubes were incubated at 65 °C overnight for reverse cross‐linking. The next day, 300 μL DNA extraction buffer was added to each tube and DNA was extracted as described (Paterson *et al*., 1993). The resulting DNA was dissolved in 26 μL ddH_2_O and take 1 μL for measuring concentration by NanoDrop.

For biotin‐SA purification of biotinylated fragments, a total of 10–20 μL SA magnetic beads (10 mg/mL, e.g. Sera‐Mag Streptavidin‐Blocked Magnetic SpeedBeads, Cytiva) were washed twice with 500 μL 1× Western blot (WB) buffer (5 mm Tris–HCl, pH7.5, 0.5 mm EDTA, 1 m NaCl, 0.02% Tween‐20), once with 500 μL 2× WB buffer and finally resuspended in 50 μL of 2× WB buffer (25 μL each for a two‐step purification). We performed a two‐step purification for each sample: The DNA sample (25 μL in ddH_2_O) was first mixed with 25 μL SA magnetic beads in 2× WB buffer in a PCR tube, and incubated at 37 °C for 20 min in the PCR machine without heat lid. After incubation, place the tubes in a magnetic stand for 5 min to collect the beads. Take a second PCR tube containing the supernatant from first PCR tube (50 μL) and the remaining beads (collected on a magnetic stand before the supernatant from the first PCR tube was added) and incubate once more at 37 °C for 20 min. The resulting beads from two tubes were combined together and washed twice with 500 μL 1× WB buffer, once with 500 μL 10 mm Tris buffer (pH 8.0) and finally resuspended in 50 μL ddH_2_O.

Libraries for deep sequencing were constructed by on‐beads PCR, with the PCR reaction (100 μL) set up as follows: 50 μL beads with affinity purified DNA, 20 μL ddH_2_O, 20 μL of 5× TruePrep Amplify Enzyme Buffer (Vazyme, Nanjing, Jiangsu, China), 4 μL of 10 uM N50X Primer, 4 μL of 10 μm N70X Primer and 2 μL of TruePrep Amplify Enzyme (Vazyme). The adaptor pooling guide strategy from Illumina was followed. Each PCR solution was mixed gently and 100 μL mineral oil added on top to prevent evaporation. PCR was performed as described (Tao *et al*., 2020), starting with a low cycle number and adding 2 cycles as needed until visible PCR bands were obtained on agarose gel analysis. After PCR, the SA beads were removed and PCR products purified using commercial DNA purification beads (e.g. AMPure XP beads). Finally, the library was redissolved in 25 μL ddH_2_O for quality control (QC) and deep sequencing.

For B‐CUT&Tag‐qPCR, we performed the B‐CUT&Tag procedure as described above, except that after DNA extraction, the DNA was redissolved in 30 μL ddH_2_O, from which 5 μL was taken out for use as ‘input’ in a subsequent qPCR assay. The remaining 25 μL was subjected to biotin‐SA purification of biotinylated fragments as described above. The DNA‐bound SA beads were finally resuspended in 50 μL 30 mm NaOAc pH 9.0 and 95% formamide, then incubated at 90°C for 10 min to release the DNA. A magnetic stand was again used to collect the supernatant (~50 μL). qPCR reactions were performed using 1 μL purified DNA and 1 μL input DNA as templates and performed in triplicate. The gene‐specific qPCR primers were designed according to the same criteria as used with ChIP‐qPCR, which was designed to cover ~250 bp sequences flanking the putative TF‐binding sites.

The relative proportion of target content was calculated using the ∆Ct method, taking the Ct values of the IgG sample as control. That is, the Ct values of the B‐CUT&Tag antibody sample were normalized to the Ct values of the IgG control, ∆Ct = Ct_Ab_ – Ct_IgG_. Fold enrichment of the target region in each antibody sample was then determined relative to the IgG control: Fold enrichment = 2^−∆Ct^. Alternatively, the Ct values of each CUT&Tag sample, whether antibody or IgG, can be normalized using its corresponding input as control: ∆Ct = Ct_sample_ – Ct_input_. The percentage of input for each B‐CUT&Tag sample is then calculated as: Percent input = 2^−∆Ct^ × 100%.

### Chromatin immunoprecipitation (ChIP)

ChIP was performed as described (Haring *et al*., 2007) with modifications. Briefly, cross‐linking and nuclei isolation were performed as described in ‘B‐CUT&Tag‐seq & B‐CUT&Tag‐qPCR’ part above. For AtSPL9 ChIP‐seq assay using the same amount of nuclei as that for AtSPL9 B‐CUT&Tag‐seq, 100 μL nuclei (equals ~20 μg chromatin) isolated from 1 g cross‐linked tissue was resuspended in 320 μL ice‐cold nuclei lysis buffer (50 mm Tris pH 8.0, 10 mm EDTA, 0.4% w/v SDS, 0.1% protease inhibitor cocktail), chromatin sonication was then performed with M220 Focused‐ultrasonicator (Covaris) using the following setups: Peak Power 75.0, Duty Factor 20.0, Cycles/Brust 200, Avg. Power 15.0, for a total of 10 min. After sonication, the clear supernatant, which contains sonicated chromatin was collected after centrifuge at 12 000 ***g*** at 4°C for 10 min and was adjusted to 420 μL using ice‐cold nuclei lysis buffer. A 400 μL aliquot of sonicated chromatin was frozen in liquid nitrogen and stored at −80°C for further use after checking the sonication efficiency. To check the efficiency of sonication, the remaining 20 μL aliquot of sonicated chromatin was mixed with 560 μL TE buffer (10 mm Tris pH 8.0, 1 mm EDTA), 20 μL 5 m NaCl and 20 μL 20% SDS, reverse cross‐linking was performed at 65 °C overnight and the DNA was purified using phenol/chloroform method as described (Tao *et al*., 2020) and was dissolved in 50 μL ddH_2_O. Five microlitre of the resulting DNA was analysed by 2% agarose gel electrophoresis, the chromatin was sheared to a length between 200 bp and 600 bp. For immunoprecipitation, the reaction was setup by adding 100 μL of sonicated chromatin, 900 μL ChIP Ab incubation buffer (50 mm Tris pH 8.0, 1 mm EDTA, 0.1% v/v Triton X‐100, 150 mm NaCl, 10 μg/mL BSA), 20 μL Protein A + G magnetic beads (CAT#16–663; Millpore) and 2 μL normal mouse IgG or anti‐FLAG antibody. Set two tubes each for IgG control and anti‐FLAG antibody (equals ~5 μg chromatin/reaction) and incubated the tubes overnight at 4 °C with gentle agitation (Rotator at 12 rpm). After immunoprecipitation, the washing, elution, reverse cross‐linking steps were performed as described (Haring *et al*., 2007). After reverse cross‐linking, the DNA was purified using phenol/chloroform method as described (Tao *et al*., 2020) and was dissolved in 30 μL ddH_2_O. The resulting DNA was used for NGS or qPCR.

### mRNA‐seq

A total of 1 μg RNA per sample was used as input material for RNA sample preparations. Sequencing libraries were generated using the NEBNext® Ultra™ II RNA Library Prep Kit for Illumina® (NEB, Ipswich, MA, USA) following the manufacturer's recommendations, and index codes were added to attribute sequences to each sample. Three biological replicates were set up for each inflorescence sample from 28‐day‐old *pSPL9::3xflag‐rSPL9* and WT plants.

### Bioinformatic analysis

Bioinformatic analysis of mRNA‐seq data was performed as described (Pertea *et al*., 2016). Bioinformatic analysis of B‐CUT&Tag‐seq data and ChIP‐seq data were likewise performed as described for CUT&Tag‐seq (Tao *et al*., 2020). Only uniquely mapped and de‐duplicated reads were used for peak calling by macs2 (*q‐*value cut‐off 0.05).

### Accession numbers

All data supporting the findings of this study are available in the article or its Supplementary Information or from the corresponding author upon reasonable request. Source data generated in this study, including the raw data from B‐CUT&Tag, CUT&Tag and mRNA‐seq, have been deposited in the National Center for Biotechnology Information Sequence Read Archive under the accession number PRJNA746662.

## Conflict of interest

The authors have declared that no competing interests exist.

## Author contributions

X.T., S.X. and J.W. conceived the project and designed the experiments. X.T. performed the experiments. X.T., S.X., J.W., X.G., G.H., Y.H., J.W., F.X., S.F. and G.C. analysed the data and interpreted the results. X.T., S.X. and J. W. wrote the manuscript.

## Supporting information


**Figure S1** Formaldehyde cross‐linking is applicable for the following B‐CUT&Tag reaction.
**Figure S2** Agarose gel electrophoresis analysis results from *SPL9* inflorescences B‐CUT&Tag.
**Figure S3** AtSPL9 ChIP‐seq using the same amount of nuclei for AtSPL9 CUT&Tag‐seq showed low‐quality data.
**Figure S4** GO enrichment analysis of B‐CUT&Tag peak‐related genes.
**Figure S5** The scenario 1 and scenario 2 products accounted for the majority of the tagmentation products.
**Figure S6** The on‐beads extension by DNA polymerase before DNA retrieval reduced the non‐specific amplification by P1 primer.
**Figure S7** B‐CUT&Tag‐qPCR using primer P1/gene‐specific primer as primer pairs in the reaction showed consistent results.
**Table S1** Oligos used in this study.
**Table S2** NGS sequencing metadata.
**Table S3** List of 233 DEGs (Foldchange >1.50 or <0.67, *P* < 0.05) identified from mRNA‐seq that also have B‐CUT&Tag peaks (peak fold_enrichment >2).
**Table S4** Selected DEGs having B‐CUT&Tag peaks that are involved in multiple biological processes.

## References

[pbi14029-bib-0001] Abe, H. , Urao, T. , Ito, T. , Seki, M. , Shinozaki, K. and Yamaguchi‐Shinozaki, K. (2003) Arabidopsis AtMYC2 (bHLH) and AtMYB2 (MYB) function as transcriptional activators in abscisic acid signaling. Plant Cell 15, 63–78. 12509522 10.1105/tpc.006130 PMC143451

[pbi14029-bib-0002] Bartosovic, M. , Kabbe, M. and Castelo‐Branco, G. (2021) Single‐cell CUT&Tag profiles histone modifications and transcription factors in complex tissues. Nat Biotechnol. Nat. Biotechnol. 39, 825–835. 33846645 10.1038/s41587-021-00869-9 PMC7611252

[pbi14029-bib-0003] Borsani, O. , Zhu, J. , Verslues, P.E. , Sunkar, R. and Zhu, J.K. (2005) Endogenous siRNAs derived from a pair of natural cis‐antisense transcripts regulate salt tolerance in *Arabidopsis* . Cell 123, 1279–1291. 16377568 10.1016/j.cell.2005.11.035 PMC3137516

[pbi14029-bib-0004] Cho, S.K. , Ryu, M.Y. , Seo, D.H. , Kang, B.G. and Kim, W.T. (2011) The Arabidopsis RING E3 ubiquitin ligase AtAIRP2 plays combinatory roles with AtAIRP1 in Abscisic acid‐mediated drought stress responses. Plant Physiol. 157, 2240–2257. 21969385 10.1104/pp.111.185595 PMC3327188

[pbi14029-bib-0005] Cui, L.G. , Shan, J.X. , Shi, M. , Gao, J.P. and Lin, H.X. (2014) The miR156‐SPL9‐DFR pathway coordinates the relationship between development and abiotic stress tolerance in plants. Plant J. 80, 1108–1117. 25345491 10.1111/tpj.12712

[pbi14029-bib-0006] Dong, Z. , Xiao, Y. , Govindarajulu, R. , Feil, R. , Siddoway, M.L. , Nielsen, T. , Lunn, J.E. *et al*. (2019) The regulatory landscape of a core maize domestication module controlling bud dormancy and growth repression. Nat. Commun. 10, 3810. 31444327 10.1038/s41467-019-11774-w PMC6707278

[pbi14029-bib-0007] Fu, Y. , Ma, H. , Chen, S. , Gu, T. and Gong, J. (2018) Control of proline accumulation under drought via a novel pathway comprising the histone methylase CAU1 and the transcription factor ANAC055. J. Exp. Bot. 69, 579–588. 29253181 10.1093/jxb/erx419 PMC5853435

[pbi14029-bib-0008] Furey, T.S. (2012) ChIP–seq and beyond: new and improved methodologies to detect and characterize protein–DNA interactions. Nat. Rev. Genet. 13, 840–852. 23090257 10.1038/nrg3306 PMC3591838

[pbi14029-bib-0009] Gou, J.Y. , Felippes, F.F. , Liu, C.J. , Weigel, D. and Wang, J.W. (2011) Negative regulation of anthocyanin biosynthesis in *Arabidopsis* by a miR156‐targeted SPL transcription factor. Plant Cell 23, 1512–1522. 21487097 10.1105/tpc.111.084525 PMC3101539

[pbi14029-bib-0010] Haring, M. , Offermann, S. , Danker, T. , Horst, I. , Peterhansel, C. and Stam, M. (2007) Chromatin immunoprecipitation: optimization, quantitative analysis and data normalization. Plant Methods 3, 11. 17892552 10.1186/1746-4811-3-11 PMC2077865

[pbi14029-bib-0011] Jakobsen, J.S. , Braun, M. , Astorga, J. , Gustafson, E.H. , Sandmann, T. , Karzynski, M. , Carlsson, P. *et al*. (2007) Temporal ChIP‐on‐chip reveals Biniou as a universal regulator of the visceral muscle transcriptional network. Genes Dev. 21, 2448–2460. 17908931 10.1101/gad.437607 PMC1993875

[pbi14029-bib-0012] Jia, H. , Ren, H. , Gu, M. , Zhao, J. , Sun, S. , Zhang, X. , Chen, J. *et al*. (2011) The Phosphate Transporter Gene OsPht1;8 Is Involved in Phosphate Homeostasis in Rice. Plant Physiol. 156, 1164–1175. 21502185 10.1104/pp.111.175240 PMC3135946

[pbi14029-bib-0013] Kaya‐Okur, H.S. , Wu, S.J. , Codomo, C.A. , Pledger, E.S. , Bryson, T.D. , Henikoff, J.G. , Ahmad, K. *et al*. (2019) CUT&Tag for efficient epigenomic profiling of small samples and single cells. Nat. Commun. 10, 1930. 31036827 10.1038/s41467-019-09982-5 PMC6488672

[pbi14029-bib-0014] Kaya‐Okur, H.S. , Janssens, D.H. , Henikoff, J.G. , Ahmad, K. and Henikoff, S. (2020) Efficient low‐cost chromatin profiling with CUT&Tag. Nat. Protoc. 15, 3264–3283. 32913232 10.1038/s41596-020-0373-x PMC8318778

[pbi14029-bib-0015] Kong, Y. , Wang, G. , Chen, X. , Li, L. , Zhang, X. , Chen, S. , He, Y. *et al*. (2021) OsPHR2 modulates phosphate starvation‐induced OsMYC2 signalling and resistance to *Xanthomonas oryzae* pv. oryzae. Plant Cell Environ. 44, 3432–3444. 33938007 10.1111/pce.14078

[pbi14029-bib-0016] Li, R.J. , Li, L.M. , Liu, X.L. , Kim, J.C. , Jenks, M.A. and Lu, S. (2019) Diurnal regulation of plant epidermal wax synthesis through antagonistic roles of the transcription factors SPL9 and DEWAX. Plant Cell 31, 2711–2733. 31484683 10.1105/tpc.19.00233 PMC6881124

[pbi14029-bib-0017] Lian, H. , Wang, L. , Ma, N. , Zhou, C.M. , Han, L. , Zhang, T.Q. and Wang, J.W. (2021) Redundant and specific roles of individual MIR172 genes in plant development. PLoS Biol. 19, e3001044. 33529193 10.1371/journal.pbio.3001044 PMC7853526

[pbi14029-bib-0018] Liu, F. , Wang, Z. , Ren, H. , Shen, C. , Li, Y. , Ling, H.Q. , Wu, C. *et al*. (2010) OsSPX1 suppresses the function of OsPHR2 in the regulation of expression of OsPT2 and phosphate homeostasis in shoots of rice. Plant J. 62, 508–517. 20149131 10.1111/j.1365-313X.2010.04170.x

[pbi14029-bib-0019] Lu, Y. , Ronald, P.C. , Han, B. , Li, J. and Zhu, J.‐K. (2020) Rice Protein Tagging Project: A Call for International Collaborations on Genome‐wide In‐Locus Tagging of Rice Proteins. Mol. Plant 13, 1663–1665. 33189907 10.1016/j.molp.2020.11.006

[pbi14029-bib-0020] Magome, H. , Yamaguchi, S. , Hanada, A. , Kamiya, Y. and Oda, K. (2008) The DDF1 transcriptional activator upregulates expression of a gibberellin‐deactivating gene, GA2ox7, under high‐salinity stress in *Arabidopsis* . Plant J. 56, 613–626. 18643985 10.1111/j.1365-313X.2008.03627.x

[pbi14029-bib-0021] Mao, Y.B. , Liu, Y.Q. , Chen, D.Y. , Chen, F.Y. , Fang, X. , Hong, G.J. , Wang, L.J. *et al*. (2017) Jasmonate response decay and defense metabolite accumulation contributes to age‐regulated dynamics of plant insect resistance. Nat. Commun. 8, 13925. 28067238 10.1038/ncomms13925 PMC5233801

[pbi14029-bib-0022] Ouyang, W. , Zhang, X. , Peng, Y. , Zhang, Q. , Cao, Z. , Li, G. and Li, X. (2021) Rapid and low‐input profiling of histone marks in plants using nucleus CUT&Tag. Front. Plant Sci. 12, 634679. 33912205 10.3389/fpls.2021.634679 PMC8072009

[pbi14029-bib-0023] Paterson, A.H. , Brubaker, C.L. and Wendel, J.F. (1993) A rapid method for extraction of cotton (*Gossypium* spp.) genomic DNA suitable for RFLP or PCR analysis. Plant Mol. Biol. Report. 11, 122–127.

[pbi14029-bib-0024] Pertea, M. , Kim, D. , Pertea, G.M. , Leek, J.T. and Salzberg, S.L. (2016) Transcript‐level expression analysis of RNA‐seq experiments with HISAT, StringTie and Ballgown. Nat. Protoc. 11, 1650–1667. 27560171 10.1038/nprot.2016.095 PMC5032908

[pbi14029-bib-0025] Qiao, J. , Zhang, Z. , Ji, S. , Liu, T. , Zhang, X. , Huang, Y. , Feng, W. *et al*. (2022) A distinct role of STING in regulating glucose homeostasis through insulin sensitivity and insulin secretion. Proc. Natl. Acad. Sci. USA 119, e2101848119. 35145023 10.1073/pnas.2101848119 PMC8851542

[pbi14029-bib-0042] Reznikoff, W.S. (2003) Tn5 as a model for understanding DNA transposition. Molecular microbiology 47, 1199–1206. 12603728 10.1046/j.1365-2958.2003.03382.x

[pbi14029-bib-0026] Rubio, V. , Linhares, F. , Solano, R. , Martín, A.C. , Iglesias, J. , Leyva, A. and Paz‐Ares, J. (2001) A conserved MYB transcription factor involved in phosphate starvation signaling both in vascular plants and in unicellular algae. Genes Dev. 15, 2122–2133. 11511543 10.1101/gad.204401 PMC312755

[pbi14029-bib-0027] Shi, J. , Zhao, B. , Zheng, S. , Zhang, X. , Wang, X. , Dong, W. , Xie, Q. *et al*. (2021) A phosphate starvation response‐centered network regulates mycorrhizal symbiosis. Cell 184, 5527–5540.e5518. 34644527 10.1016/j.cell.2021.09.030

[pbi14029-bib-0028] Smaczniak, C. , Immink, R.G. , Muino, J.M. , Blanvillain, R. , Busscher, M. , Busscher‐Lange, J. , Dinh, Q.D. *et al*. (2012) Characterization of MADS‐domain transcription factor complexes in *Arabidopsis* flower development. Proc. Natl. Acad. Sci. U. S. A. 109, 1560–1565. 22238427 10.1073/pnas.1112871109 PMC3277181

[pbi14029-bib-0029] Spitz, F. and Furlong, E.E.M. (2012) Transcription factors: from enhancer binding to developmental control. Nat. Rev. Genet. 13, 613–626. 22868264 10.1038/nrg3207

[pbi14029-bib-0030] Swift, J. and Coruzzi, G.M. (2017) A matter of time—How transient transcription factor interactions create dynamic gene regulatory networks. Biochim Biophys Acta Gene Regul Mech 1860, 75–83. 27546191 10.1016/j.bbagrm.2016.08.007 PMC5203810

[pbi14029-bib-0031] Tao, X. , Feng, S. , Zhao, T. and Guan, X. (2020) Efficient chromatin profiling of H3K4me3 modification in cotton using CUT&Tag. Plant Methods 16, 120. 32884577 10.1186/s13007-020-00664-8 PMC7460760

[pbi14029-bib-0032] Thalhammer, A. and Hincha, D.K. (2014) A mechanistic model of COR15 protein function in plant freezing tolerance: integration of structural and functional characteristics. Plant Signal. Behav. 9, e977722. 25496049 10.4161/15592324.2014.977722 PMC4623512

[pbi14029-bib-0033] Ucar, D. , Beyer, A. , Parthasarathy, S. and Workman, C.T. (2009) Predicting functionality of protein‐DNA interactions by integrating diverse evidence. Bioinformatics 25, i137–i144. 19477979 10.1093/bioinformatics/btp213 PMC2687967

[pbi14029-bib-0034] Vokes, S.A. , Ji, H. , Wong, W.H. and McMahon, A.P. (2008) A genome‐scale analysis of the cis‐regulatory circuitry underlying sonic hedgehog‐mediated patterning of the mammalian limb. Genes Dev. 22, 2651–2663. 18832070 10.1101/gad.1693008 PMC2559910

[pbi14029-bib-0035] Wang, J.W. , Czech, B. and Weigel, D. (2009) miR156‐regulated SPL transcription factors define an endogenous flowering pathway in *Arabidopsis thaliana* . Cell 138, 738–749. 19703399 10.1016/j.cell.2009.06.014

[pbi14029-bib-0036] Wu, S.J. , Furlan, S.N. , Mihalas, A.B. , Kaya‐Okur, H.S. , Feroze, A.H. , Emerson, S.N. , Zheng, Y. *et al*. (2021) Single‐cell CUT&Tag analysis of chromatin modifications in differentiation and tumor progression. Nat. Biotechnol. 39, 819–824. 33846646 10.1038/s41587-021-00865-z PMC8277750

[pbi14029-bib-0037] Xie, Y. , Liu, Y. , Ma, M. , Zhou, Q. , Zhao, Y. , Zhao, B. , Wang, B. *et al*. (2020) *Arabidopsis* FHY3 and FAR1 integrate light and strigolactone signaling to regulate branching. Nat. Commun. 11, 1955. 32327664 10.1038/s41467-020-15893-7 PMC7181604

[pbi14029-bib-0038] Xu, T. , Xiao, M. and Yu, L. (2021) Method for efficient soluble expression and purification of recombinant hyperactive Tn5 transposase. Protein Expr. Purif. 183, 105866. 33716122 10.1016/j.pep.2021.105866

[pbi14029-bib-0039] Yu, N. , Cai, W.J. , Wang, S. , Shan, C.M. , Wang, L.J. and Chen, X.Y. (2010) Temporal control of trichome distribution by microRNA156‐targeted SPL genes in *Arabidopsis thaliana* . Plant Cell 22, 2322–2335. 20622149 10.1105/tpc.109.072579 PMC2929091

[pbi14029-bib-0040] Zhang, C. , Li, C. , Liu, J. , Lv, Y. , Yu, C. , Li, H. , Zhao, T. *et al*. (2017) The OsABF1 transcription factor improves drought tolerance by activating the transcription of COR413‐TM1 in rice. J. Exp. Bot. 68, 4695–4707. 28981779 10.1093/jxb/erx260 PMC5853872

[pbi14029-bib-0041] Zhang, Q.Q. , Wang, J.G. , Wang, L.Y. , Wang, J.F. , Wang, Q. , Yu, P. , Bai, M.Y. *et al*. (2020) Gibberellin repression of axillary bud formation in *Arabidopsis* by modulation of DELLA‐SPL9 complex activity. J. Integr. Plant Biol. 62, 421–432. 31001922 10.1111/jipb.12818

