# Supplementary material for: Biotinylated Tn5 transposase‐mediated CUT&Tag efficiently profiles transcription factor‐DNA interactions in plants
Source: Plant Biotechnol J. 2023 Mar 2;21(6):1191–205. doi: 10.1111/pbi.14029 (PMC10214755; doi:10.1111/pbi.14029)
Supplement: Supplementary file 1 — Figure S1 Formaldehyde cross‐linking is applicable for the following B‐CUT&Tag reaction. Figure S2 Agarose gel electrophoresis analysis results from SPL9 inflorescences B‐CUT&Tag. Figure S3 AtSPL9 ChIP‐seq using the same amount of nuclei for AtSPL9 CUT&Tag‐seq showed low‐quality data. Figure S4 GO enrichment analysis of B‐CUT&Tag peak‐related genes. Figure S5 The scenario 1 and scenario 2 products accounted for the majority of the tagmentation products. Figure S6 The on‐beads extension by DNA polymerase before DNA retrieval reduced the non‐specific amplification by P1 primer. Figure S7 B‐CUT&Tag‐qPCR using primer P1/gene‐specific primer as primer pairs in the reaction showed consistent results. Table S1 Oligos used in this study. Table S2 NGS sequencing metadata. Table S3 List of 233 DEGs (Foldchange >1.50 or <0.67, P < 0.05) identified from mRNA‐seq that also have B‐CUT&Tag peaks (peak fold_enrichment >2). Table S4 Selected DEGs having B‐CUT&Tag peaks that are involved in multiple biological processes. [file PBI-21-1191-s001.pdf]

Supplemental Figure 1

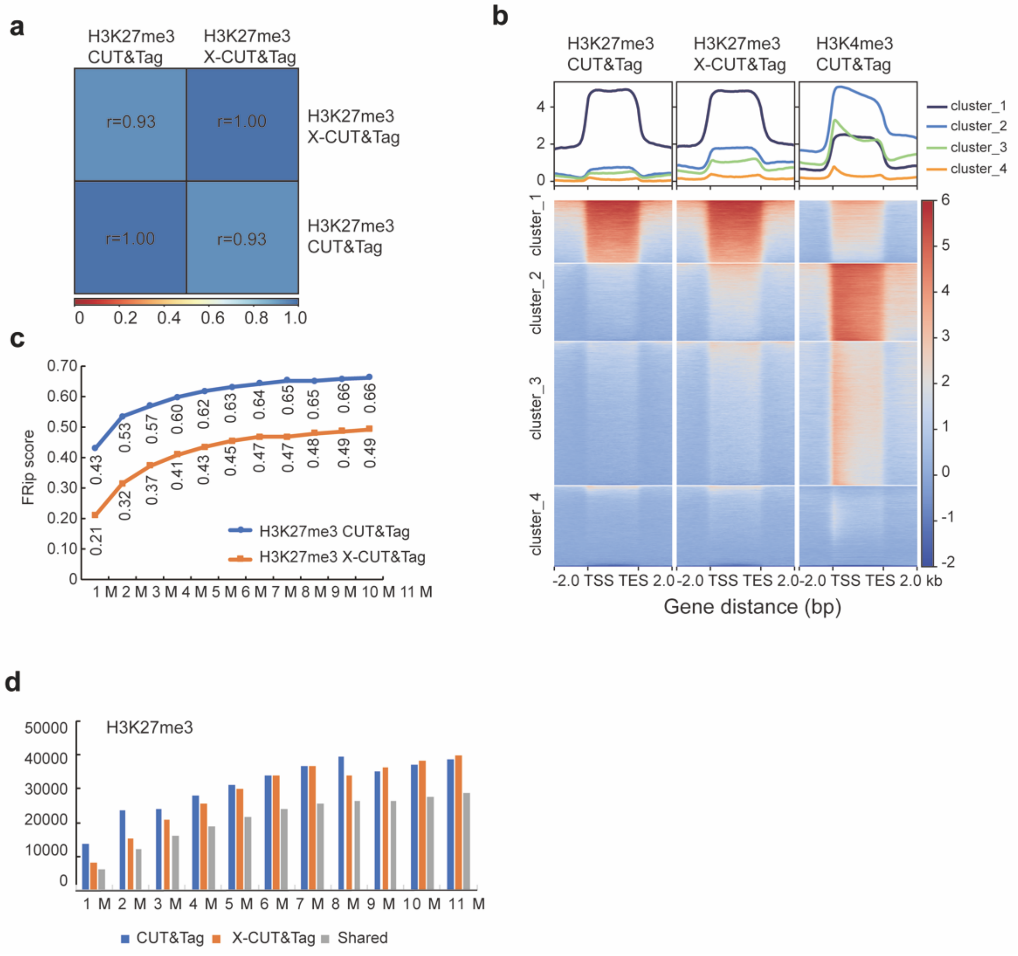

Figure S1. Formaldehyde crosslinking is applicable for the following B-CUT&Tag reaction. (a) Hierarchically clustered correlation matrix of native CUT&Tag and cross-linking CUT&Tag (X-CUT&Tag) on H3K27me3 profiling in cotton leaves. X-CUT&Tag was performed using cotton nuclei pre-treated with 1% formaldehyde for 5 min. Pearson's r was indicated. (b) Heatmap clustering of H3K27me3 signals near protein coding genes that generated by native CUT&Tag and X-CUT&Tag. H3K4me3 profiling using the same nuclei from cotton leaves was provided for comparison. A trend was discovered that chromatin with higher H3K27me3 modification showed lower H3K4me3 levels (e.g. genes in cluster 1), and vice versa (genes in cluster 2), indicating that CUT&Tag is applicable not only for active chromatin marker but also suitable for silent chromatin marker. (c) FRiP (fraction of all mapped reads that fall into peak regions) scores under different sequencing depth (from 1 M to 11 M clean reads) showed the efficiency of peak-calling between native CUT&Tag and X-CUT&Tag. (d) Number of called peaks of native CUT&Tag, X-CUT&Tag and their shared ones under different sequencing depth.

Supplemental Figure 2

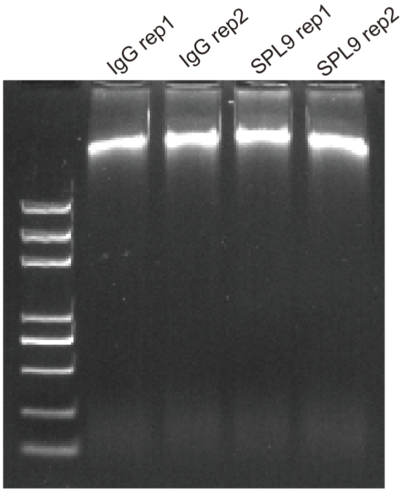

Figure S2. Agarose gel electrophoresis analysis results from *SPL9* inflorescences B-CUT&Tag. Figure showed unbound fraction of chromatin after biotin-SA based purification.

Supplemental Figure 3

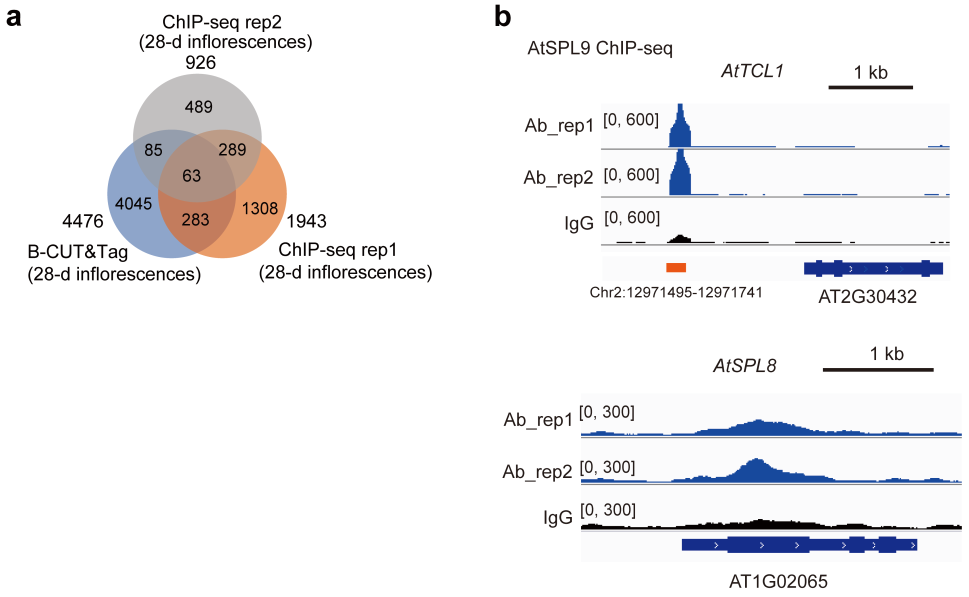

Figure S3. AtSPL9 ChIP-seq using the same amount of nuclei for AtSPL9 CUT&Tag-seq showed low-quality data. (a) Interactive venn graph showed overlapped potential target genes from two replicates of AtSPL9 ChIP-seq and AtSPL9 B-CUT&Tag-seq using inflorescences from 28-day-old plants. (b) IGV profiles of AtSPL9 CUT&Tag-seq data depicting binding profiles of AtSPL9 at gene loci including *AtTCL1* and *AtSPL8*. The binding of AtSPL9 to the orange box-indicated region in the promoter of *AtTCL1* were previously confirmed by ChIP-qPCR (Yu et al., 2010).

Supplemental Figure 4

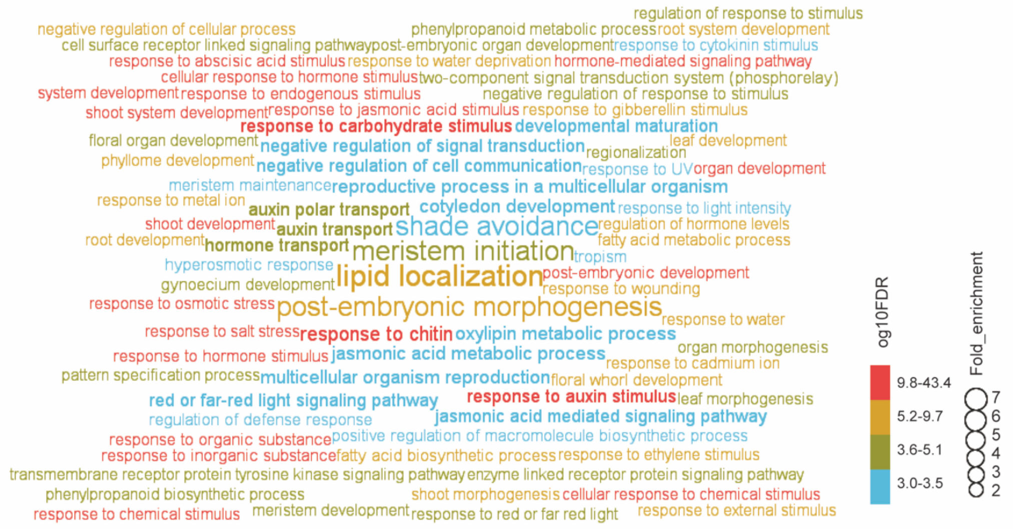

Figure S4. GO enrichment analysis of B-CUT&Tag peak-related genes. Word cloud showed 80 GO terms with more than 2-fold enrichment compared with background. Word size represents foldchange, word color indicates significance(-$\log_{10}$FDR).

Supplemental Figure 5

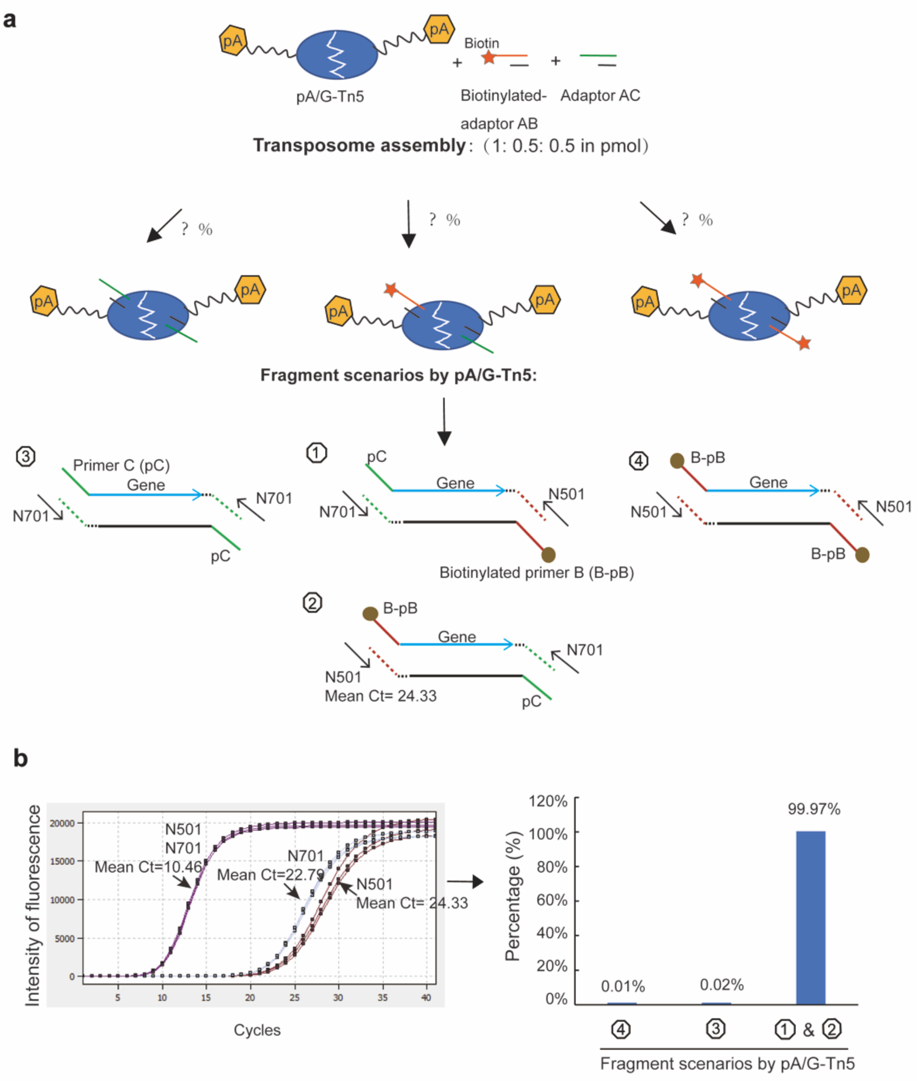

Figure S5. The scenario 1 and scenario 2 products accounted for the majority of the tagmentation products. (a) pA-Tn5 transposome homo-dimer assembly and four scenarios for adaptor "pasting" after tagmentation. The adaptor primers in pA-Tn5 transposome homo-dimer, including primer C(pC) and Biotinylated primer B (B-pB) were indicated. **(b)** qPCR amplification curves and the normalized proportion of different tagmentation scenarios. qPCR was performed in in triplicate using pA-Tn5 tagmentation products from 1 μg *Arabidopsis* genomic DNA as templates. The PCR primers, N501 and N701, which were used for determining different tagmentation scenarios were indicated in pannel a.

Supplemental Figure 6

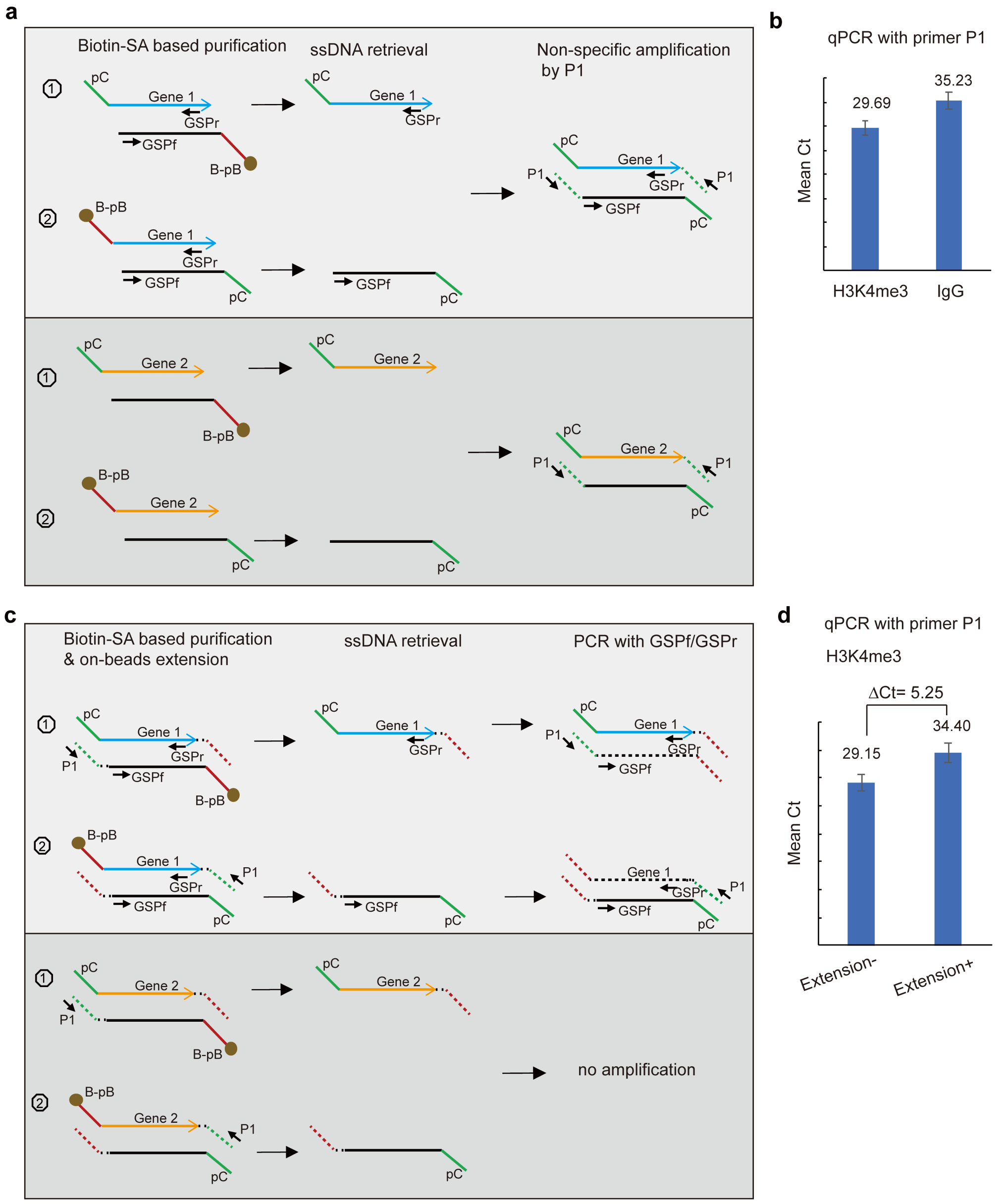

Figure S6. The on-beads extension by DNA polymerase before DNA retrieval reduced the non-specific amplification by P1 primer. a Schematic diagram showed the formation of dsRNA by single-strand DNA (ssDNA) that retrieved from scenario 1 and scenario 2 (as shown in Figure S6a), which has complementary or partially complementary sequences to each other, as indicated, P1 also works to amplify fragments from Gene 2, thus lead to non-specific priming. **b** qPCR results showed average Ct values of H3K4me3 sample and the IgG control using single P1 primer. **c** Schematic diagram indicated the on-beads extension step by DNA polymerase before ssDNA retrieval significantly reduced the non-specific amplification by P1 primer. **d** qPCR results showed average Ct values of H3K4me3 B-CUT&Tag samples without extension

(-) or with extension (+) step before ssDNA retrieval followed with qPCR using single P1 primer.

Supplemental Figure 7

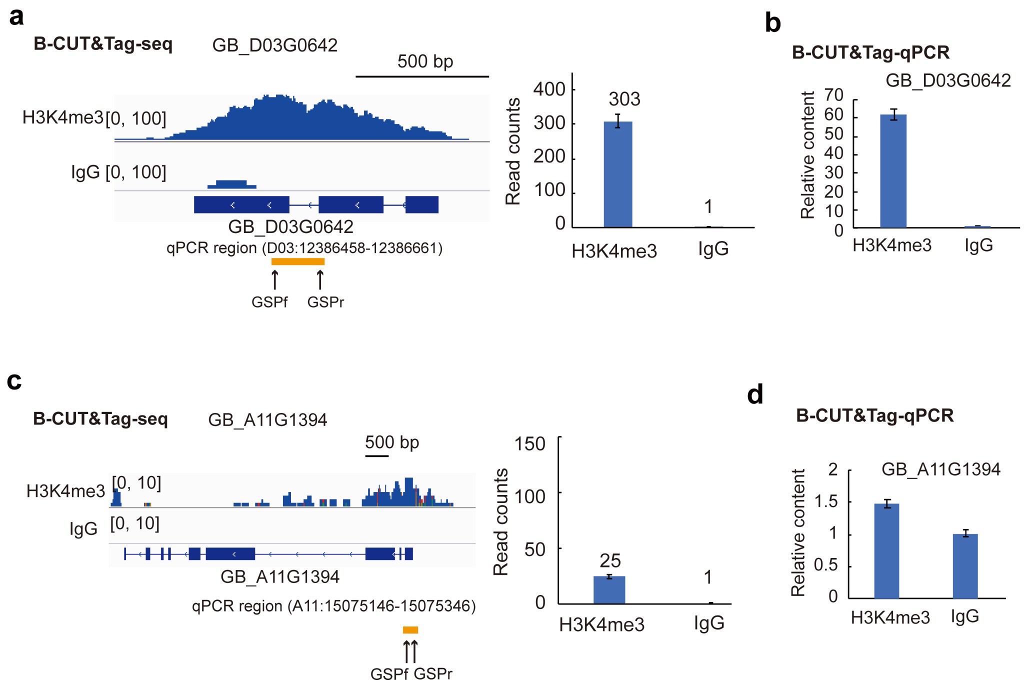

Figure S7. B-CUT&Tag-qPCR using primer P1/gene-specific primer as primer pairs in the reaction showed consistent results. (a) IGV profiles depicting the H3K4me3 modification status at *GB_D03G0642* gene loci compard with IgG control. The number of reads uniquely mapped to the region between GSPf/GSPr were summarized. (b) Normalized qPCR results showed relative content of fragments using GSPf/GSPr as primer pairs in the H3K4me3 sample compared with IgG control. (c) IGV profiles depicting the H3K4me3 modification status at *GB_A11G1394* gene loci, the number of reads uniquely mapped to the indicated region between GSPf/GSPr were summarized. (b) Normalized qPCR results showed relative content of fragments using GSPf/GSPr as primer pairs in the H3K4me3 sample compared with IgG control.

Supplemental Table 1. Oligos used in this study.

Name	Purification Method	Sequences （5'-3'）	Purpose
Primer A	HPLC	5'-phos-CTGTCTCTTATACACATCT-NH2 -3' (5'-Phosphate, 3'-AminolinkerC7)	Tn5 adaptor
Primer B	DSL	TCGTCGGCAGCGTCAGATGTGTATAAGAGACAG (5'-Biotin TEG)	Tn5 adaptor
Primer C	DSL	GTCTCGTGGGCTCGGAGATGTGTATAAGAGACAG	Tn5 adaptor
N501	DSL	AATGATACGGCGACCACCGAGATCTACACTAGATCGCTCGTCGGCAGCGTC	Index Primers
N502	DSL	AATGATACGGCGACCACCGAGATCTACACCTCTCTATTCGTCGGCAGCGTC	Index Primers
N503	DSL	AATGATACGGCGACCACCGAGATCTACACTATCCTCTTCGTCGGCAGCGTC	Index Primers
N504	DSL	AATGATACGGCGACCACCGAGATCTACACAGAGTAGATCGTCGGCAGCGTC	Index Primers
N505	DSL	AATGATACGGCGACCACCGAGATCTACACGTAAGGAGTCGTCGGCAGCGTC	Index Primers
N506	DSL	AATGATACGGCGACCACCGAGATCTACACACTGCATATCGTCGGCAGCGTC	Index Primers
N507	DSL	AATGATACGGCGACCACCGAGATCTACACAAGGAGTATCGTCGGCAGCGTC	Index Primers
N508	DSL	AATGATACGGCGACCACCGAGATCTACACCTAAGCCTTCGTCGGCAGCGTC	Index Primers
N701	DSL	CAAGCAGAAGACGGCATACGAGATTAAGGCGAGTCTCGTGGGCTCGG	Index Primers

N702	DSL	CAAGCAGAAGACGGCATACGAGATCGTACTAGGTC TCGTGGGCTCGG	Index Primers
N703	DSL	CAAGCAGAAGACGGCATACGAGATAGGCAGAAGT CTCGTGGGCTCGG	Index Primers
N704	DSL	CAAGCAGAAGACGGCATACGAGATTCCTGAGCGTC TCGTGGGCTCGG	Index Primers
N705	DSL	CAAGCAGAAGACGGCATACGAGATGGACTCCTGTC TCGTGGGCTCGG	Index Primers
N706	DSL	CAAGCAGAAGACGGCATACGAGATTAGGCATGGTC TCGTGGGCTCGG	Index Primers
N707	DSL	CAAGCAGAAGACGGCATACGAGATCTCTCTACGTC TCGTGGGCTCGG	Index Primers
N708	DSL	CAAGCAGAAGACGGCATACGAGATCAGAGAGGGT CTCGTGGGCTCGG	Index Primers
N709	DSL	CAAGCAGAAGACGGCATACGAGATGCTACGCTGTC TCGTGGGCTCGG	Index Primers
N710	DSL	CAAGCAGAAGACGGCATACGAGATCGAGGCTGGT CTCGTGGGCTCGG	Index Primers
N711	DSL	CAAGCAGAAGACGGCATACGAGATAAGAGGCAGT CTCGTGGGCTCGG	Index Primers
N712	DSL	CAAGCAGAAGACGGCATACGAGATGTAGAGGAGT CTCGTGGGCTCGG	Index Primers
P1	DSL	CGTACTAGGTCTCGTGGGCTCGG	qPCR, overlap with Primer C
GSPf_GB_D10 G1774_R1	DSL	CAAATGGGGTCGTCGGCGGT	qPCR, GB_D10G1774, Region 1, forward
GSPr_GB_D10 G1774_R1	DSL	AGTGGATTTCCCAGGCGGCCTTA	qPCR, GB_D10G1774, Region 1, reverse
GSPf_GB_D10 G1774_R2	DSL	TTTGGGTCGCGCCTATCCACT	qPCR, GB_D10G1774, Region 2, forward

GSPr_GB_D10G1774_R2	DSL	AGGGGAAAAATTAAGCACCAGTCGC	qPCR, GB_D10G1774, Region 2, reverse
GSPf_GB_D03G0642	DSL	TGATGATCTCCATCGCCACTCCT	qPCR, GB_D03G0642, forward
GSPr_GB_D03G0642	DSL	TGCTCAAGGCTGTCACTGGTGG	qPCR, GB_D03G0642, reverse
GSPf_GB_A11G1394	DSL	CCTCCTCATCAAAGATGGCATCAGCA	qPCR, GB_A11G1394, forward
GSPr_A11G1394	DSL	TCGGCATAGTAGCTGAGAAAGCATCA	qPCR, GB_A11G1394, reverse
GSPf_AtTCL	DSL	GAGAAATCAAATCGAGGGCGTA	qPCR, AtTCL1(AT2G30432), forward
GSPr_AtTCL	DSL	AACCCCTAGATTGTTGAAAGTTGAA	qPCR, AtTCL1(AT2G30432), reverse
GSPf_AtTRY	DSL	TCTTTCGCCGTAGACTACAGAT	qPCR, AtTRY(AT5G53200), forward
GSPr_AtTRY	DSL	GACCCATTAAACTAACGTATTTAAT	qPCR, AtTRY(AT5G53200), reverse
GSPf_AtFUL	DSL	AAAAACTTGTCTCCATGCAAAAAG	qPCR, AtFUL(AT5G60910), forward
GSPr_AtFUL	DSL	TTGTCGAGTCCTCATTGGCTACT	qPCR, AtFUL(AT5G60910), reverse
GSPf_AtACT2	DSL	CTTCTTCCGCTCTTTCTTTCCAAGGT	qPCR, AtACT2 (AT3G18780), forward
GSPf_AtACT2	DSL	TGGATCTCTCCATCAAGGTCAAGCCA	qPCR, AtACT2 (AT3G18780), reverse
GSPf_OsPT2	DSL	ACAGCGATCCCCTCTGCTCT	qPCR, OsPT2(LOC_Os03g05640.1), sense
GSPr_OsPT2	DSL	GGGGTGTGTCCATTTTTAATGCGT	qPCR, OsPT2(LOC_Os03g05640.1), anti-sense
GSPf_OsPT8	DSL	CGCGTCCATGGCTGACAGG	qPCR, OsPT8(LOC_Os10g30790.1), sense

GSPr_OsPT8	DSL	TCTGGTGACGTGTTGGGACC	qPCR,<br>OsPT8(LOC_Os10g30790.1),<br>anti-sense
GSPf_Os<br>RAM1	DSL	ACCATCAGCCACGGCTAAAAT	qPCR,<br>OsRAM1(LOC_Os11g31100.1),<br>sense
GSPr_Os<br>RAM1	DSL	AGAATGGCCCCATGGATTGGC	qPCR,<br>OsRAM1(LOC_Os11g31100.1),<br>anti-sense

Supplemental Table 2. Metadata of NGS sequencing of CUT&Tag libraries.

**Material**	**Sample name**	**Clean Reads**	**Unique mapped reads**	**Deduplicated unique mapped reads**	**Unique mapped reads in Peaks**
Arabidopsis WT (Col-0), leaves	AtWTIgG	1,028,555	615,497	234,074	26,390
Arabidopsis WT (Col-0), leaves	AtWTH3K4me3CutTag	16,925,361	15,359,610	9,393,985	6,245,408
Arabidopsis WT (Col-0), leaves	AtWTH3K4me3BCutTagbeads-	27,282,015	24,587,214	14,700,910	9,716,263
Arabidopsis WT (Col-0), leaves	AtWTH3K4me3BCutTag	35,803,819	26,146,755	13,408,868	8,694,982
Arabidopsis pSPL9::3xflag-rSPL9, inflorescence	AtIgGBCutTaginflo_rep1	14,210,532	11,172,989	5,413,033	1,410,873
Arabidopsis pSPL9::3xflag-rSPL9, inflorescence	AtIgGBCutTaginflo_rep2	21,015,709	16,486,059	7,981,648	2,087,392
Arabidopsis pSPL9::3xflag-rSPL9, inflorescence	AtSPL9BCutTaginflo_rep1	18,517,274	16,236,982	9,876,358	4,188,268
Arabidopsis pSPL9::3xflag-rSPL9, inflorescence	AtSPL9BCutTaginflo_rep2	17,512,705	15,143,135	9,316,303	3,913,513

Arabidopsis pSPL9::3xflag-rSPL9 plants, aerial parts of 21-day-old plants	AtSPL9BCutTag21d	17,301,503	12,215,054	4,214,245	1,493,073

Supplemental Table 3. List of 233 DEGs (90 up and 143 down) from mRNA-seq that with B-CUT&Tag peaks. DEGs with fold_enrichment > 2 from B-CUT&Tag peaks were listed.

**Up-regulated DEGs**	**Foldchange >1.50 (pSPL9::3xflag-rSPL9 vs. WT) （90 genes）**	***P* value cutoff (<0.05)**	**Down-regulated DEGs （143 genes）**	**Foldchange < 0.67 (pSPL9::3xflag-rSPL9 vs. WT)**	***P* value cutoff (<0.05)**
AT4G38820	72.3347274	0.01849046	AT1G12320	0.66982808	0.00060874
AT5G58750	12.9292611	0.00014753	AT1G68520	0.66899198	4.9516E-06
AT2G42200	7.19971564	0	AT1G13950	0.66790284	0.00747751
AT5G05250	3.81255269	7.5107E-32	AT3G56200	0.66545471	1.0241E-05
AT5G52390	3.6496623	1.712E-157	AT5G17220	0.6649738	8.965E-07
AT1G54040	3.34150779	3.656E-105	AT4G36950	0.66455106	0.00317941
AT4G15210	2.76971671	8.323E-169	AT4G35900	0.66333157	0.04330817
AT5G39090	2.69651912	4.5897E-26	AT4G30650	0.66326447	9.583E-12
AT2G18300	2.54508072	0.00403874	AT3G14850	0.66269895	0.01742482
AT1G06340	2.47694213	8.575E-08	AT2G31010	0.66236327	7.9766E-08
AT2G44840	2.42964777	0.00012298	AT2G47190	0.66167439	0.00709855
AT4G27280	2.3455744	7.7282E-32	AT5G06720	0.66166658	0.00321381
AT4G17340	2.2760243	2.4909E-33	AT1G03940	0.66079552	0.0001014
AT5G16350	2.21827387	2.9022E-18	AT5G64260	0.66063757	1.3783E-20
AT5G61600	2.17296997	1.5722E-12	AT2G17290	0.65664093	1.1002E-08
AT5G07990	2.1625494	5.4278E-73	AT5G12940	0.6560802	2.1128E-12
AT3G10600	2.15847357	0.00712186	AT5G01520	0.65557568	2.142E-07
AT4G26950	2.04329991	2.6051E-06	AT5G01540	0.65545487	0.0002133
AT3G50280	2.01236574	3.2029E-32	AT2G42610	0.65520675	0.04933154
AT4G31870	1.98883411	2.5346E-14	AT3G55100	0.65509848	0.00123538
AT1G01060	1.9618631	2.3404E-25	AT1G75450	0.65468272	9.2174E-07
AT3G10560	1.90356886	3.7445E-05	AT3G16400	0.65347718	2.4885E-05
AT3G22840	1.87258552	2.2795E-38	AT5G13210	0.65292661	0.04887956

AT1G72920	1.86883865	6.6433E-07	AT1G63860	0.65281511	0.02319566
AT1G61120	1.85537782	3.3752E-06	AT1G51940	0.65237443	1.4727E-13
AT4G16590	1.84519624	1.2894E-43	AT3G53800	0.65126979	6.0397E-05
AT1G72490	1.83412314	0.02149308	AT1G51805	0.65077455	1.5709E-13
AT3G25905	1.83165974	3.9215E-05	AT1G02450	0.65033467	0.00014237
AT5G62730	1.82820852	5.9271E-29	AT2G45180	0.65027179	1.7924E-12
AT1G69260	1.81799026	1.5416E-06	AT4G02330	0.64641778	4.0774E-06
AT5G14570	1.81491458	2.2263E-24	AT4G22790	0.64285804	0.00038275
AT1G80050	1.78963356	1.2423E-11	AT4G38550	0.64234052	2.0493E-09
AT3G01840	1.77804964	0.0007727	AT2G44490	0.64186414	9.0364E-13
AT3G09390	1.77803544	4.4278E-26	AT2G35710	0.64152046	0.01450379
AT2G40970	1.77725214	0.00016496	AT2G37370	0.6403565	0.03088924
AT1G05680	1.76824901	7.8843E-23	AT4G33050	0.63913856	3.3437E-09
AT1G72940	1.76791367	4.8861E-09	AT1G02400	0.63871927	0.02301435
AT4G34930	1.76666088	0.01912111	AT1G19380	0.63864861	0.00902145
AT5G20670	1.76401538	7.641E-09	AT4G21870	0.63824282	2.6502E-05
AT1G01590	1.74518185	2.4459E-08	AT5G47220	0.63699151	0.00730295
AT1G53887	1.74347913	1.6302E-05	AT3G15500	0.63689115	2.2621E-06
AT5G17030	1.73738738	5.3025E-15	AT5G02200	0.63654172	0.00609184
AT1G56150	1.72522308	0.02999161	AT5G64120	0.63641813	1.0154E-07
AT3G27170	1.72518347	0.00862313	AT5G07010	0.63639907	6.4739E-07
AT4G35090	1.70876625	1.42E-21	AT4G25420	0.63279448	0.00015025
AT4G10290	1.70019085	0.00304598	AT5G61590	0.62928716	2.4885E-05
AT1G49640	1.69960046	0.00603016	AT5G24470	0.62781701	2.9357E-06
AT4G28270	1.69293564	8.9427E-05	AT1G05000	0.62534655	0.0458357
AT3G50060	1.68852144	2.9011E-17	AT5G17490	0.62439199	0.00031197
AT5G22630	1.68617326	1.2812E-32	AT1G80120	0.62354585	6.0751E-06
AT1G26770	1.68107067	8.8642E-28	AT5G06870	0.62338075	2.494E-07
AT3G01500	1.67537175	3.4023E-32	AT1G29660	0.6229435	1.0159E-12

AT3G53950	1.67223269	8.8074E-17	AT4G13790	0.62284879	0.00083313
AT4G01060	1.66639385	8.1803E-09	AT4G38840	0.62140826	7.4272E-06
AT5G53200	1.654623	1.9745E-10	AT5G15230	0.62107477	3.286E-20
AT2G37170	1.64951078	1.1227E-20	AT4G16563	0.62081402	2.7966E-05
AT3G62410	1.64048808	1.9914E-11	AT5G47240	0.62039372	1.7092E-12
AT5G23730	1.63682526	4.7764E-07	AT2G39180	0.61669686	4.9937E-05
AT5G13170	1.62291122	1.2109E-19	AT4G16146	0.61411364	4.1646E-08
AT4G21930	1.62131799	2.214E-06	AT4G18880	0.60925567	0.00342867
AT3G50560	1.61405644	8.9975E-10	AT1G78260	0.60887462	0.00025148
AT1G17100	1.60759541	9.6637E-17	AT1G69040	0.60876168	5.0129E-20
AT5G54160	1.60223274	4.716E-27	AT1G78450	0.60800883	0.00148729
AT5G55250	1.59766981	3.199E-15	AT4G11320	0.60457281	2.37E-30
AT4G27250	1.58925766	0.01135837	AT5G08760	0.60363446	0.00172472
AT5G19730	1.58513443	2.399E-12	AT4G30074	0.60020463	0.02961586
AT2G40100	1.58448556	6.9569E-31	AT5G65390	0.597189	9.6512E-07
AT3G51240	1.58421442	1.4804E-10	AT1G02813	0.59469142	4.4241E-15
AT3G19680	1.58209606	1.8536E-15	AT3G57780	0.59402203	0.00027836
AT4G33810	1.57934663	0.00177838	AT4G37800	0.59224559	1.9797E-22
AT5G18840	1.57443806	0.00661722	AT1G12090	0.59005782	1.0214E-36
AT3G18950	1.57172015	0.00122848	AT5G18010	0.58988804	0.01254618
AT4G28250	1.56558795	1.4328E-24	AT3G09032	0.58640302	0.02532506
AT5G51970	1.56450895	2.9705E-14	AT2G42885	0.58394038	2.0044E-08
AT4G36830	1.55716033	0.03360664	AT1G76590	0.58261659	0.01238303
AT4G26530	1.5533227	3.0909E-13	AT4G15250	0.58196299	0.03614585
AT3G16175	1.55194731	0.03184365	AT5G20230	0.58057139	7.7871E-27
AT1G22990	1.54817847	0.00264444	AT3G10040	0.57656344	5.5007E-08
AT3G06145	1.54773536	0.00499862	AT2G41180	0.57444594	0.02221982
AT5G22460	1.5467518	5.8264E-15	AT3G06070	0.57363439	0.00102293
AT5G01880	1.54307419	1.2497E-08	AT2G24600	0.57068818	0.00050617

AT4G39480	1.54155934	1.1752E-20	AT5G53290	0.56814704	0.00926105
AT3G56360	1.52281874	1.0347E-10	AT4G39830	0.56682564	0.00389577
AT3G48510	1.51735488	0.01510321	AT5G61440	0.56654026	4.5077E-14
AT5G57350	1.51680026	2.9371E-10	AT5G62520	0.5658404	0.03861617
AT1G07440	1.51549684	2.5849E-15	AT2G39360	0.56308991	1.6007E-08
AT3G12410	1.51513804	4.2963E-05	AT1G72600	0.55918884	2.2321E-06
AT3G22800	1.50669198	0.00012533	AT1G14700	0.55896378	2.5148E-05
AT5G47610	1.50384779	0.03260844	AT2G46530	0.55819556	5.0873E-06
AT2G26080	1.50319024	4.6894E-08	AT1G80840	0.55749959	0.0048588
			AT4G34970	0.55675264	4.0235E-07
			AT2G42530	0.55298336	1.2433E-25
			AT5G49130	0.55043999	2.4726E-22
			AT1G08890	0.54749829	1.2231E-05
			AT3G10020	0.54270946	7.2204E-20
			AT3G14020	0.5344445	7.4271E-07
			AT5G50950	0.5334776	1.0953E-21
			AT5G54510	0.53293621	1.0172E-22
			AT3G02550	0.51817325	1.582E-28
			AT5G19190	0.51627241	1.0173E-06
			AT1G72416	0.51605055	4.43E-06
			AT3G62150	0.50661828	2.1002E-06
			AT1G01480	0.50612345	0.00182469
			AT3G04210	0.49999527	2.0942E-27
			AT1G57990	0.49710646	2.4655E-29
			AT5G23350	0.4964144	0.0003579
			AT4G04330	0.49621667	0.01958721
			AT5G20250	0.49205793	7.0432E-31
			AT4G17490	0.485622	0.00012132
			AT1G05065	0.48167495	0.00622808

			AT3G51400	0.47558245	1.4478E-08
			AT1G18200	0.4748673	3.6239E-06
			AT5G19240	0.47435069	8.6571E-22
			AT1G30040	0.46587699	1.6522E-39
			AT3G26200	0.45211467	1.9652E-14
			AT1G73066	0.45145115	0.0384443
			AT4G27970	0.44722145	0.02133669
			AT5G62360	0.4438762	3.6242E-21
			AT4G27440	0.43524735	3.3204E-56
			AT3G15630	0.43303267	8.0404E-21
			AT4G30370	0.4268712	0.00042604
			AT2G43010	0.42242593	9.2439E-18
			AT5G03360	0.42044213	1.2397E-07
			AT4G32340	0.40803918	6.4231E-09
			AT1G22370	0.40802861	0.00293226
			AT2G41100	0.39005765	7.83E-54
			AT4G38850	0.38123814	0.0355125
			AT5G54720	0.38115479	0.00392877
			AT1G78440	0.36903086	1.3493E-24
			AT1G14880	0.3587143	0.00263957
			AT5G03350	0.35013535	3.3834E-28
			AT4G01950	0.32135798	5.3048E-13
			AT2G39855	0.31502118	1.3274E-07
			AT3G48520	0.31487362	0.00057476
			AT2G39250	0.30853356	0.03919862
			AT5G54710	0.30438351	2.0745E-27
			AT1G07050	0.29985766	0.02843362
			AT5G19040	0.29933155	6.4739E-07
			AT4G34410	0.27973014	0.02915331

			AT5G48850	0.25296032	2.9553E-15
			AT4G38825	0.21409037	4.0263E-05
			AT1G12610	0.15192214	8.9383E-06
			AT2G42440	0.01720072	0.01141676

Supplemental Table 4. Selected DEGs with B-CUT&Tag peaks that involved in multiple biological process.

**DEGs**	**Up/Down**	**GO terms**	**Description/Phenotypes**
**Growth & Development & Immunity**			
CLE27	Up	cell-cell signaling involved in cell fate commitment	CLE27 mutant showed root apical meristem (RAM) termination, slow growth rate, epinastic rosette leaf morphology
GBSS1	Up	starch biosynthetic process	ADP-glucose-starch glucosyltransferase activity
TRY, ETC3	Up	trichome branching & differentiation	Negative regulators of trichome development
HBI1	Up	pattern recognition receptor signaling pathway	negative control of plant immunity
SAI-LLP1, LecRKA4.1, TN13	Down	effector-triggered immunity response, or pattern recognition receptor signaling pathway or innate immune response	SAI-LLP1 OE plants potentiates defense to *P. syringae; tn13* mutant plants showed enhanced susceptibility to *P. syringae*
FD	Down	regulation of photoperiodism, flowering	bZIP protein required for positive regulation of flowering
**Secondary metabolism**			
F3H, TT7, OMT1	Up	flavonoid biosynthetic process	*Pro35S: MIM156* plants which has increased SPL9 activity accumulate increased levels of flavonoids
TT19	Down	anthocyanin-containing compound metabolic process	anthocyanin transporter; mutant has reduced accumulation of anthocyanins in leaves & stems
**Phytohormone catabolism & homeostasis**			
ATIAMT1	Up	auxin homeostasis	Converts IAA to MeIAA
GA2OX1, GA2OX6, GA2OX2	Down	gibberellin catabolic process	*GA2ox* gene family inactivate bioactive GA
CYP94B3	Down	jasmonic acid metabolic process	*CYP94B3* mediates inactivation of bioactive JA-Ile

GH3.6	Down	auxin homeostasis	IAA-amido synthase that conjugates Ala, Asp, Phe, and Trp to auxin
IPT5	Down	cytokinin biosynthetic process	Catalyzes the transfer of an isopentenyl group from dimethylallyl diphosphate (DMAPP) to ATP and ADP.
**Phytohormone signaling**			
RGL3	Down	gibberellic acid mediated signal	DELLA subfamily that acts as a negative regulator of GA signaling
**Abiotic stress responses**			
CA1/ DEG10	Up	response to cold	*deg10* mutant were impaired in root elongation, especially at elevated temperature, caused a decrease in seed production
CAT2	Up	response to oxidative stress	*cat2* mutant have increased H_2O_2 levels and increased H_2O_2 sensitivity
LHY	Up	response to salt stress	The *lhy*, *cca1* double mutants severely affect flowering time, light responses and circadian rhythms; LHY-OE plants had reduced levels of ABA under drought stress, whereas *lhy* mutant exhibited an altered rhythm of ABA accumulation
DIG1/AITR2	Up	negative regulation of ABA signaling	Also known as ABA-induced transcription repressors (AITR), *aitr2 aitr5 aitr6* triple mutant showed enhanced tolerance to drought and salt
DDF1	Down	response to freezing, response to heat, response to salt stress, response to water deprivation	*DDF1*-OE plants showed delayed flowering and dwarfism, reduction of GA biosynthesis, and increased tolerance to high levels of salt
COR15B	Down	response to cold	*COR15B* protein function in plant freezing tolerance
DEWAX	Down	response to water deprivation	*dewax* mutant showed enhanced wax synthesis
MYB2	Down	response to water deprivation, response to salt stress	*MYB2*-OE plants showed significant hypersensitivity to ABA; *MYB2*-OE plants showed increases stress tolerance
ANAC055	Down	response to water deprivation	*ANAC055*-OE have increased drought tolerance
SRO5	Down	response to salt stress	*sro5* mutant accumulated less proline under salt stress and was more sensitive to NaCl stress
AIRP2	Down	response to salt stress	AIRP2-OE plants were highly tolerant to severe drought stress

FITNESS	Down	cell redox homeostasis	*FITNESS* -OE plants lines showed increased levels of ROS; *FITNESS* absence significantly improved seed yield suggesting an effective fine-tuning trade-off between reproductive success and defence responses
**Shade avoidance**			
PIF4, FHL	Down	response to red or far red light	Shade (low R:FR ratios) results in progressive inactivation of phyB, leading to enhanced activity of PIF4-auxin pathway, which trigger elongation responses
**Aging**			
SAG29/SWEET15	Up	leaf senescence	OE plants exhibit accelerated senescence
**Nutrient utilization**			
MYB77	Up	response to potassium deprivation	OE plants accumulate more K under low K stress
NRT2.7	Up	nitrate transport	OE plants accumulate more nitrate in seeds
